# A deep insight into ferroptosis in lung disease: facts and perspectives

**DOI:** 10.3389/fonc.2024.1354859

**Published:** 2024-03-18

**Authors:** Fan Zhang, Yu Xiang, Qiao Ma, E. Guo, Xiansheng Zeng

**Affiliations:** ^1^ Wuhan University of Science and Technology, School of Medicine, Wuhan, China; ^2^ Xiangyang Central Hospital, Affiliated Hospital of Hubei University of Arts and Science, Xiangyang, China

**Keywords:** ferroptosis, lung diseases, iron metabolism, respiratory disorders, therapy

## Abstract

In the last decade, ferroptosis has received much attention from the scientific research community. It differs from other modes of cell death at the morphological, biochemical, and genetic levels. Ferroptosis is mainly characterized by non-apoptotic iron-dependent cell death caused by iron-dependent lipid peroxide excess and is accompanied by abnormal iron metabolism and oxidative stress. In recent years, more and more studies have shown that ferroptosis is closely related to the occurrence and development of lung diseases. COPD, asthma, lung injury, lung fibrosis, lung cancer, lung infection and other respiratory diseases have become the third most common chronic diseases worldwide, bringing serious economic and psychological burden to people around the world. However, the exact mechanism by which ferroptosis is involved in the development and progression of lung diseases has not been fully revealed. In this manuscript, we describe the mechanism of ferroptosis, targeting of ferroptosis related signaling pathways and proteins, summarize the relationship between ferroptosis and respiratory diseases, and explore the intervention and targeted therapy of ferroptosis for respiratory diseases.

## 1 Introduction

In 2003, DOLMA et al. discovered Erastin, an antioxidant that can inhibit glutathione synthesis (1). Subsequently, another compound that activates non-apoptotic cell death, RSL3, was discovered in 2008 by YAGODA et al. and YANG et al (2). In 2012, DIXON et al. officially named iron-dependent nonapoptotic cell death “Ferroptosis” (3). Since then, ferroptosis has been gradually well-known by domestic and foreign scholars, and has aroused widespread concern.

Ferroptosis is a new mode of cell death, which is morphologically, biochemically and genetically different from other forms of cell death (4). Morphologically, ferroptosis is mainly characterized by decreased mitochondrial cristae, changes in the bilateral membrane density of the mitochondrial membrane, and rupture of the outer membrane of the mitochondria, but the morphology and size of the cells remain normal, and chromatin is not condensed (3). Biochemically, ferroptosis continuously consumes intracellular glutathione, resulting in decreased glutathione peroxidase 4 (GPX4) activity, iron-dependent lipid peroxides cannot be reduced and metabolized by GPX4, and Fenton reaction occurs in the form of divalent iron resulting in mitochondrial reactive oxygen species (ROS) accumulation. ROS production leads to DNA damage, metabolic disorders, lipid peroxidation, and further promotes the development of ferroptosis (5–7). Genetically speaking, ferroptosis is a biological process regulated by multiple genes, which roughly includes: iron overload, lipid peroxide accumulation, amino acid metabolism disorders and other changes, and the specific regulatory mechanism needs further study (8).

**Figure 1:**
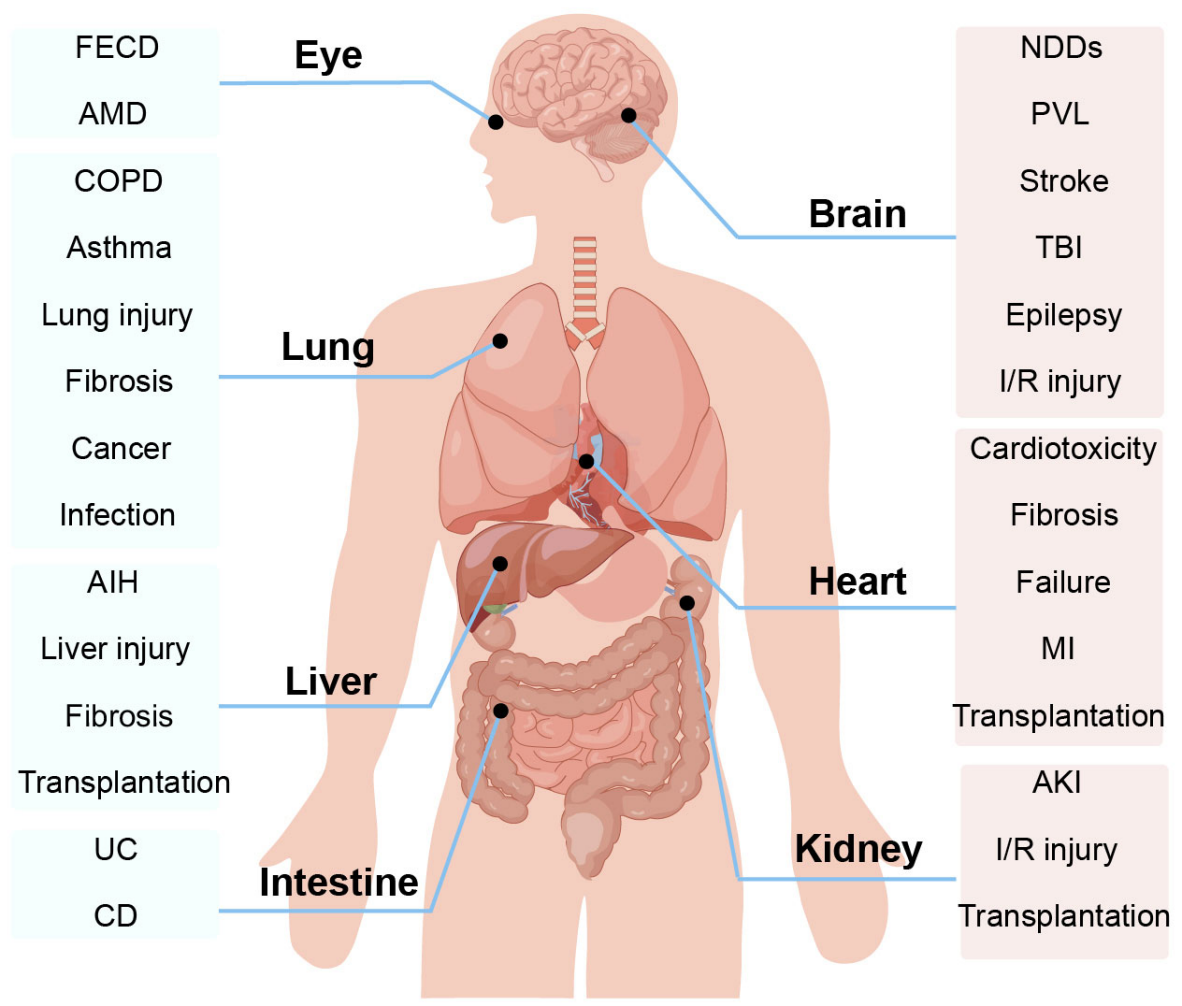
Ferroptosis is involved in the progression of a variety of organ and system diseases, such as the body’s nervous system, cardiovascular system, digestive system, and urinary system. AIH, autoimmune hepatitis; AMD, age-related macular degeneration; AKI, acute kidney injury; COPD, Chronic obstructive pulmonary disease; CD, Crohn’s disease; FECD, Fuchs’ endothelial corneal dystrophy; I/R injury, ischemia-reperfusion injury; MI, myocardial infarction; NDDs, neurodegenerative diseases; PVL, periventricular leukomalacia; TBI, traumatic brain injury.

In addition to attacking the respiratory system of the human body, ferroptosis also causes different degrees of damage to the nervous system, cardiovascular system, digestive system, and urinary system of the human body, seriously threatening human healthy life (9) (**Figure 1**). With the deepening of studies on the mechanism of ferroptosis, it has been found that ferroptosis plays a role in various biochemical reactions such as cell growth, energy metabolism, and DNA synthesis repair, which are associated with the development of lung diseases (10–12). Increasing studies have shown that ferroptosis plays a role in lung diseases by causing pathological processes such as inflammatory cell infiltration, endothelial cell damage, and disturbed cellular homeostasis (13–15). Eventually, ferroptosis can cause respiratory diseases such as chronic obstructive pulmonary disease (COPD), bronchial asthma, lung injury, pulmonary fibrosis, and lung infection (16).

In this paper, the mechanism of ferroptosis, the research progress of ferroptosis in a variety of lung diseases, and the related signaling pathways and proteins are targeted to provide new ideas and insights for the prevention and treatment of lung diseases in clinical practice (17).

## 2 Characteristics and mechanisms of ferroptosis

### 2.1 Iron overload

**Figure 2:**
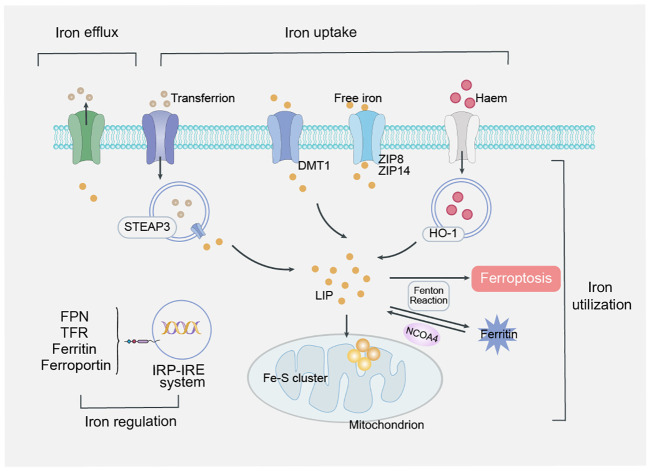
Mechanism of iron overload. Iron maintains a dynamic balance in uptake, transport, utilization and regulation, is absorbed into mucosal epithelial cells in the form of Fe^2+^, reduces Fe^3+^ in response to STEAP3, transports it to the cytoplasm through FPN, binds to ferritin and stores in the iron pool of the iron storage system. Finally, divalent metal transporter 1 (DMT1) releases iron ions from endosomes into labile iron pools in the cytoplasm. In response to H_2_O_2_, Fe^2+^ initiates a chain reaction of free radical lipid peroxidation via the Fenton reaction, ultimately leading to ferroptosis. Regulation of iron is systemic regulation of hepcidin produced by hepatic secretion and intracellular regulation of the iron regulatory protein (IRP)/iron response element (IRE) system.

Iron is an essential trace element in the body, and iron overload is a key factor in the development of ferroptosis (18). Iron maintains a dynamic balance in uptake, transport, utilization and regulation and is essential to maintain the normal physiological activities of the human body (19). Prostate six-transmembrane epithelial antigen 3 belongs to STEAP family and is an iron reductase that is able to promote the reduction of Fe^3+^ to Fe^2+^ during iron metabolism (20). Among them, FPN is the only transporter known to be intracellular iron and is present in all transport cells (21). Heme oxygenase 1 (HO-1) is a major regulator of the antioxidant system, which inhibits lipid peroxidation and protects cells from ferroptosis (22, 23) (**Figure 2**).

Regulation of iron metabolism is divided into systemic regulation by hepcidin, which is produced by hepatic secretion, and intracellular regulation by the iron regulatory protein (IRP)/iron response element (IRE) system (24, 25).In iron overload, the balance of the two major systems regulated by iron in the human body breaks, and excessive divalent iron ions induce ferroptosis by producing hydroxyl radicals through the Fenton reaction.

### 2.2 Lipid peroxidation

Lipid peroxidation plays a central role in the development of ferroptosis, and lipid peroxidation requires three steps: synthesis of membrane polyunsaturated fatty acids (PUFAs) containing phospholipid substrates, free radical priming, and enzyme induction (26).

#### 2.2.1 Phospholipid substrate synthesis

PUFAs involved in membrane phospholipid synthesis contain multiple carbon-carbon double bonds and more fragile carbon-hydrogen bonds and are therefore more sensitive to oxidation (27). It is esterified by lysophosphatidyltransferase 3 (LPCAT3) and incorporated into membrane phospholipids to form the ferroptosis lipid peroxidation substrate PUFA-PL, which turns on downstream peroxidation (28). Downregulation of LPCAT3 or ACSL4 causes substrate depletion for lipid peroxidation and reduces the risk of ferroptosis (29).

#### 2.2.2 Free radical-mediated lipid oxidation

Peroxidative degradation of PUFA-PL initiated by free radicals can be divided into three stages: initiation, propagation and termination (30). In the initial stage, labile iron, stored intracellularly as ferritin or iron-sulfur clusters, undergoes Fenton reaction and Haber-Weiss re ‐ action with hydrogen peroxide and forms hydroxyl radicals centered on oxygen with peroxyl radicals (31). In the propagation stage, LOOH captures the hydrogen atoms of adjacent lipids through free radicals and undergoes the Fenton-like reaction under the catalysis of ferrous ions, generating alkoxy free radicals (LO ·) and triggering lipid free radical chain reactions to form lipid oxidation cascades, while producing some secondary products such as malondialdehyde (MDA) and 4-hydroxynonaldehyde (4-HNE) (32). Finally, the antioxidant system is abnormal, the peroxide substrate is depleted, the cascade terminates, and causes severe damage to the cell membrane (33).

#### 2.2.3 Enzyme-mediated lipid oxidation

During the enzymatic process, the lipoxygenase (LOXs or ALOXs) family and nadph- cytochrome P450 reductase (POR) are critical to turn on lipid peroxidation during ferroptosis (34). ALOXs/LOXs are iron-containing dioxygenases that catalyze binding of free PUFAs in biofilms, promote PUFA-containing lipid oxidation, and accelerate ferroptosis progression (35).

#### 2.2.4 Deprivation of cysteine and glutathione (GSH)depletion

Cysteine is a sulfur-containing amino acid that can be synthesized endogenously via the transsulfuration pathway or acquired from extracellular cystine by cystine glutamate reverse transporter (System Xc-) (36).

Glutathione is a γ-amide bond- and thiol-containing tripeptide with antioxidant effects and integrated detoxification, and prevents lipid peroxidation in ferroptosis by providing electrons to GPX4 (37). Glutathione is stored in the human body as reduced (GSH) and oxidized (GSSG) forms and plays a key role in the antioxidant system (38). In addition to erastin-induced GSH reduction enervates GPX4 and raise ROS levels (39, 40). In addition, unlike the above system Xc- response, some cytokines can cause glutathione depletion directly by inhibiting GSH ligase, resulting in ferroptosis.

## 3 Ferroptosis-related signaling molecules and signaling pathways

**Figure 3:**
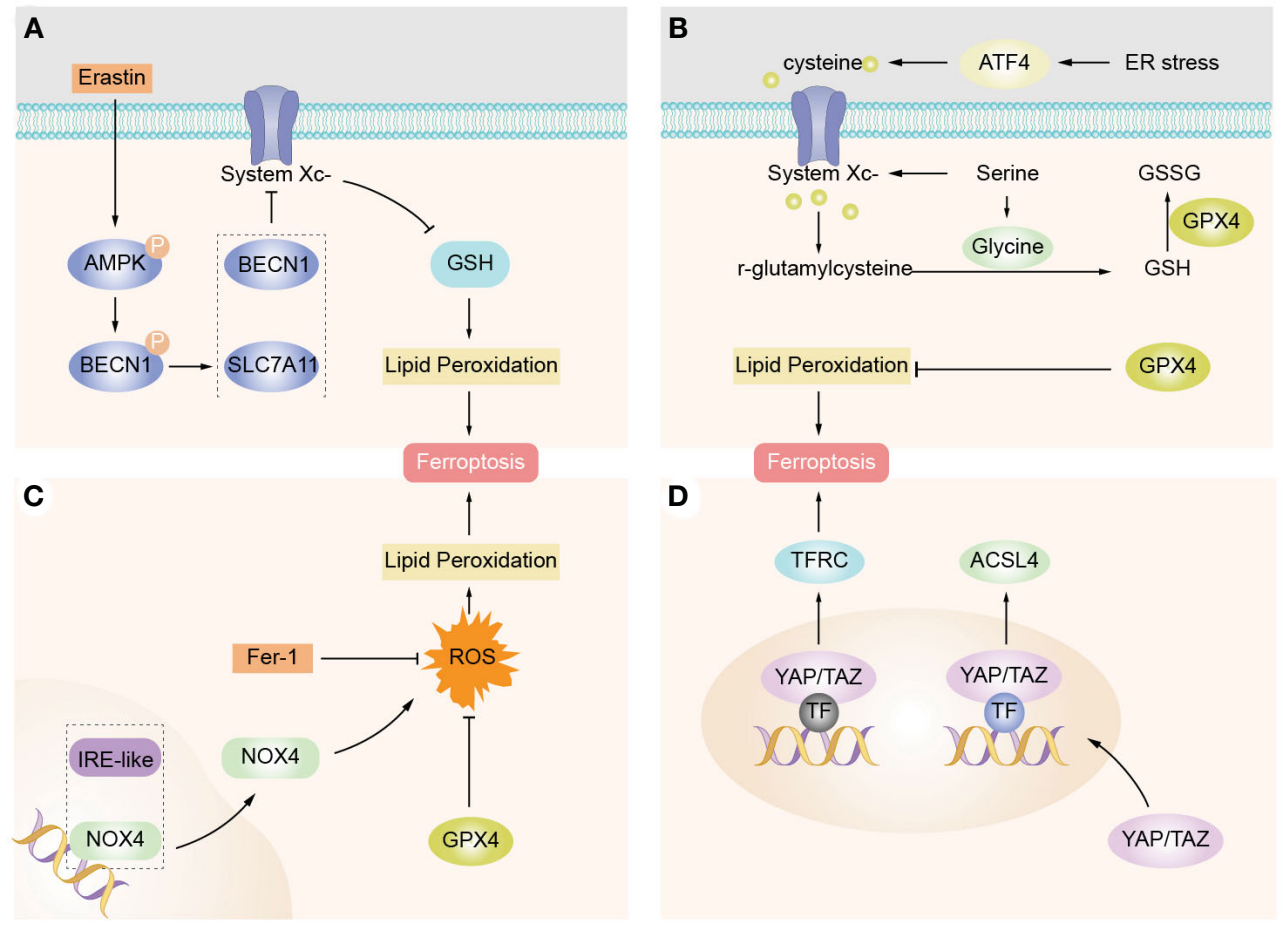
Ferroptosis-related signaling molecules and signaling pathways. **(A)** AMPK promotes complex BECN1-SLC7A11 formation mediated by AMPK, thereby inducing promotion of lipid peroxidation and ferroptosis. **(B)** ATF4 expression is elevated in cancer and promotes cell survival and tumor growth by inducing genes involved in amino acid metabolism and oxidant defense. **(C)** NOX4 is the main source of ROS production in cells and is able to inhibit intracellular ROS accumulation and protect against ferroptosis by downregulating NOX4 expression. **(D)** YAP/TAZ enters the nucleus to promote expression of EMP1, TFRC, and ACSL4, so cells are sensitive to ferroptosis.

Ferroptosis is significantly associated with the physiology and pathology of many diseases, and the related signaling pathways and regulators of ferroptosis are important ways to regulate ferroptosis. Therefore, five related pathways of ferroptosis, AMPK signaling, Activating transcription factor 4(ATF4), BECN1 signaling, NOX4 signaling, Yes associated protein/transcription costimulator with PDZ-binding domain (YAP/TAZ), will be discussed below to provide new targets for future disease therapy and new drug research (**Figure 3**).

### 3.1 AMPK signaling

It is well-known that the body maintains a dynamic balance of nutrient energy metabolism, and homeostasis will be imbalanced when the body produces stress, and glucose deprivation causes excessive ROS production leading to energy stress, indicating that glucose deprivation induces ferroptosis (41). AMPK, as an important sensor of energy metabolism in the body, opens an energy stress protection program that inhibits cellular ferroptosis by regulating the synthesis of unsaturated fatty acids (42). Overall, AMPK-induced energy stress, a protective mechanism, appears to prevent ischemia-reperfusion injury in body organs (43).

### 3.2 ATF4 signaling

ATF4, a basic leucine zipper protein, is an important factor involved in the regulation of mitochondrial stress, pathological stress, apoptosis, inflammation, related pathways, and proteins (44). Under stress conditions, the expression of ATF4 is up-regulated by phosphorylating and activating eukaryotic translation initiation factor 2a (elF2a), and ATF4 regulates gene expression after entering the nucleus, which has an effect on the development, growth, metabolic function, and oxidative response of the body. ATF4-C/EBP homologous protein (CHOP) is an important pathway to regulate pathological phenomena such as ER stress, ROS production, lipid peroxidation, and iron metabolism, and inhibition of this pathway prevents the progression of ferroptosis-related diseases such as acute lung injury (45).

### 3.3 NOX4 signaling

NOX4 is the main source of ROS production in cells, and it produces large amounts of superoxide through electron reduction reactions of NADPH. Among the NOX isoforms of the human gene, NOX4-mediated oxidative stress specifically impacts cell development and atrophy and is involved in the development of lung disease (46). By downregulating NOX4 expression, it was able to inhibit intracellular ROS accumulation, infiltration of inflammatory cells, as well as mitochondrial dysfunction, further indicating that Nox4 is involved in apoptosis through oxidative stress pathways (47). Therefore, knockdown of NOX4 factor or drug intervention may inhibit ferroptosis in cells.

### 3.4 YAP/TAZ signaling

The YAP/TAZ, as a pair of recently elucidated transcriptional regulators, is involved in the regulatory mechanism of Hippo signaling pathway and also plays an important role in cell differentiation, tissue homeostasis, organ development, and cancer development in the body (48). The expression of YAP/TAZ has been demonstrated in a variety of tumors and is significantly associated with anti-tumor therapy, clinical prognosis (49).

## 4 Ferroptosis defense pathways

**Figure 4:**
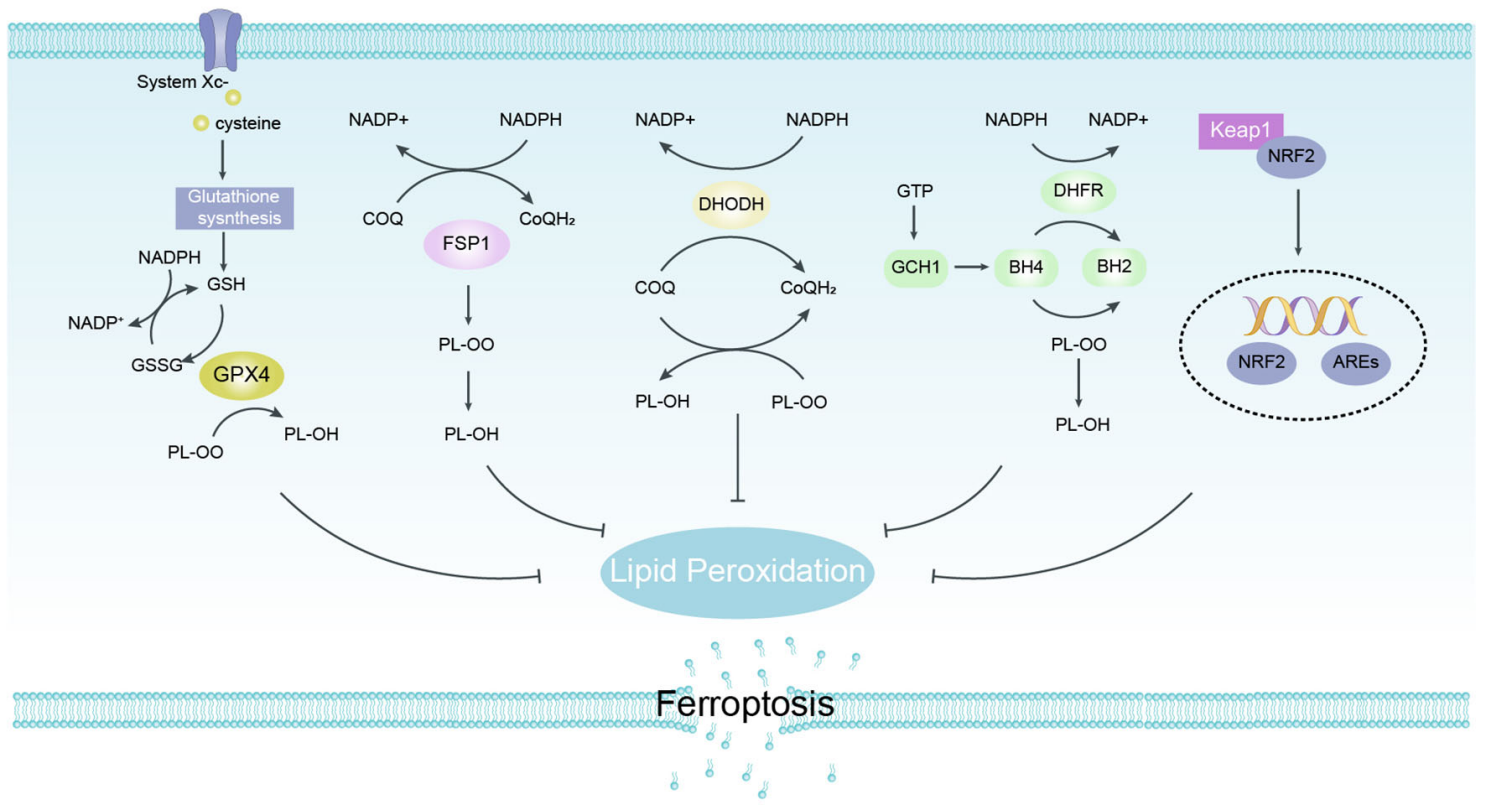
Defense Pathways of Ferroptosis. GPX4 specifically catalyzes the loss of oxidative activity of lipid peroxides in a glutathione (GSH)-dependent manner and is thought to be a major factor in the progression against ferroptosis. FSP1 does not rely on glutathione to convert ubiquinone to reduced ubiquinone on cell membranes and can inhibit peroxidation and protect against ferroptosis. DHODH traps free radicals to inhibit lipid peroxidation in mitochondria by regulating dihydroubiquinone production in the inner mitochondrial membrane. The GCH1/BH4 pathway acts as an endogenous antioxidant pathway, and GCH1 mainly protects cells from ferroptosis through the antioxidant effect of BH4. NRF2 protects body cells from ferroptosis by up-regulating multiple signals through antioxidant effects. FSP1, ferroptosis suppressor protein 1; GPX4, glutathione peroxidase 4.

Among the protective mechanisms against peroxidative damage, GSH/GPX4 axis is considered to be a major factor in the progression against ferroptosis, while non-GPX4-dependent antioxidant pathways also play an important role in the regulation of ferroptosis (**Figure 4**).

### 4.1 GSH/GPX4 axis

GSH, as the main free radical scavenger in cells, maintains biosynthesis from three amino acids, Cys), glycine, and glutamate, and resists ferroptosis progression and lipid peroxidation (50). Glutathione peroxidase 4 (GPX4) is an antioxidant enzyme containing selenocysteine (Ser) that uses GSH as a substrate to catalyze the reduction of PLOOH to non-toxic PLs-alcohol (PL-OH) and prevent the occurrence of ferroptosis using its unique catalytic ability (51, 52). The System Xc- is the core precursor of GSH synthesis and an important cofactor for GPX4 to scavenge membrane lipid peroxides and reduce oxidative stress, which plays a key role in inducing the development of ferroptosis (53).

### 4.2 NAD(P)H/FSP1-CoQ axis

Ferroptosis suppressor protein 1 (FSP1), formerly known as AIFM2, is a flavoprotein that was identified to be closely associated with ferroptosis and independent of the GPX4-GSH pathway (54). FSP1 is modified by N-terminal myristoylation and targets a variety of cell membrane structures including cytoplasmic membranes, Golgi apparatus, and perinuclear structures, and mutating the myristoyl modification site of FSP1 loses its FSP1-mediated anti- ferroptosis function (55). As NAD(P) H-dependent ubiquinone (CoQ) oxidoreductase, FSP1 inhibits the occurrence of lipid peroxidation by reducing the incomplete oxidation product of CoQ/ubiquinone to ubiquinol (CoQH2), while indirectly promoting α-tocopherol regeneration (vitamin E, a natural fat-soluble antioxidant) and jointly resisting ferroptosis.

### 4.3 GCH1/BH4/DHFR axis

Guanosine triphosphate cyclohydrolase 1 (GCH1) exerts its powerful anti-gpx4 inhibitory effect on ferroptosis by activating tetrahydrobiopterin (BH4) and dihydrobiopterin (BH2) (56). GCH1 selectively prevented the consumption of phospholipids containing two polyunsaturated fatty acid acyl tails, and increased BH4/BH2 to inhibit lipid peroxidation and iron ion denaturation (57). Another mechanism by which BH4 inhibits ferroptosis reduces CoQ to CoQH2 to enhance resistance to ferroptosis. Supplementation of BH2 *in vitro* promotes regeneration and protects cells from ferroptosis through the turnover of BH4 (58). In addition, higher levels of GCH1 were also detected in a large number of lung cancer tissue samples.

### 4.4 Mitochondria DHODH

DHODH, located on the inner mitochondrial membrane, is able to catalyze pyrimidine nucleotide synthesis, and its loss of activity leads to the accumulation of lipid peroxides in mitochondria and triggers the development of ferroptosis in GPX4-low expressing cells (59). DHODH inhibits lipid peroxidation by converting mitochondrial CoQ to CoQH2, unlike FSP1 localized outside the membrane, ectopic expression of FSP1 does not protect cells from ferroptosis (60). The application of the DHODH inhibitor brequinar inhibited the growth of tumor cells with low GPX4 expression *in vitro*, and the combined treatment of brequinar and the SLC7A11 inhibitor sulfasalazine abolished the growth of tumor cells with high GPX4 expression (61).

### 4.5 Keap1-Nrf2-ARE axis

Nuclear factor erythroid 2-related factor 2 (NRF2) is a transcription factor involved in cellular oxidative responses that advance iron storage, curb iron uptake, limits ROS production, and ultimately regulates ferroptosis in the activated state (62, 63). In addition to its role in iron metabolism, NRF2 regulates basal and inducible glutathione synthesis expression of SLC7A11 and γ-glutamylcysteine synthetase (γ-GCS) to protect against ferroptosis. Additionally, NRF2 accelerates the progression of ferroptosis by increasing Fe^2+^ from the labile iron pool by regulating heme oxygenase 1 (HO-1) (64, 65).

## 5 Small-molecule modulators of ferroptosis

### 5.1 Small molecule inducers of ferroptosis

RSL3 and erastin are small molecule compounds that induce ferroptosis in tumor cells with mutations in the oncogene RAS (66, 67). RSL3 acts by inhibiting the enzymatic activity of GPX4 and irreversibly inactivates GPX4, in which Altretamine and Withaferin A as anti-tumor drugs can directly inhibit GPX4-mediated ferroptosis in tumor cells and provide new strategies for anti-tumor therapy (68, 69).

### 5.2 Small molecule inhibitors of ferroptosis

Inhibitors of ferroptosis act by inhibiting lipid peroxidation and iron accumulation. Fer-1 and Lip-1 act as aromatic amine antioxidants and are able to prevent lipid ROS accumulation and inhibit ferroptosis (70). By downregulating 5-lipoxygenase (5-LOX), zileuton and N-acetylcysteine protect cells from lipid peroxidation caused by reactive oxygen species generation (71, 72). ACSL4 esterifies free fatty acids and is a key enzyme in regulating lipid composition. Rosiglitazone, pioglitazone, and troglitazone specifically inhibited ACSL4 activity to prevent ferroptosis and cellular lipid oxidation (73). In addition, another sign of ferroptosis is iron overload, and the iron chelators desferrioxamine (DFP) have the effect of inhibiting ferroptosis.

### 5.3 Regulation of ferroptosis by natural compounds

Recently, an increasing number of natural products have been isolated from natural resources as reagents for drug development for the treatment and prevention of diseases (74). These natural compounds are able to maintain effects such as iron homeostasis in the body, which are associated with inhibition of ferroptosis. Recently, it has been shown that artemisinin and its derivatives (artesunate, dihydroartemisinin) can not only treat malaria, but also induce iron apoptosis in cancer cells through a series of reactions (75, 76). Baicalein, discovered in a natural product library screen, is a natural ferroptosis inhibitor that inhibits ferroptosis by inhibiting erastin induction and 12/15-LOX activity.

## 6 Ferroptosis in pulmonary disease

**Figure 5:**
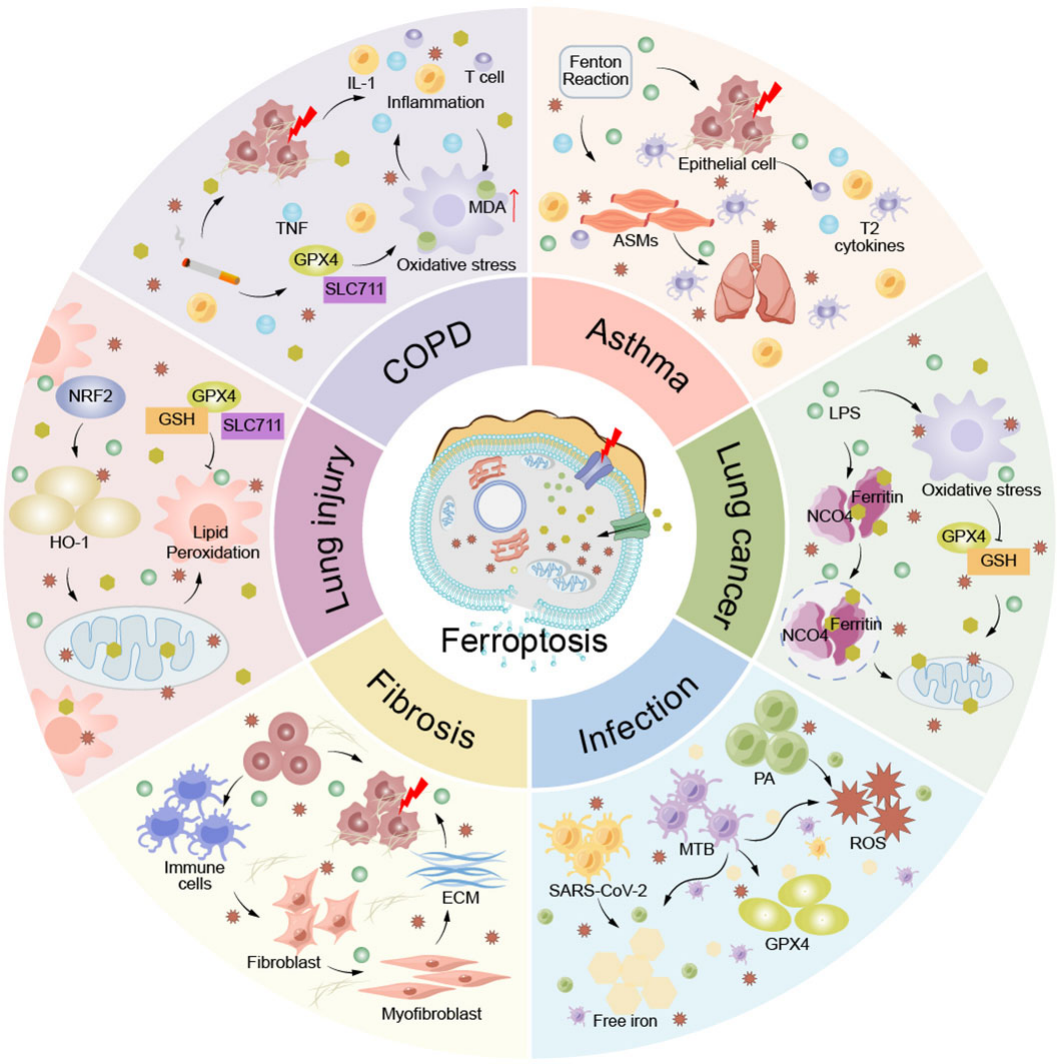
Ferroptosis in pulmonary disease. Ferroptosis plays an important role in the pathogenesis of lung diseases such as COPD, asthma, lung injury, lung cancer, pulmonary fibrosis, and lung infection. ACMs, airway smooth muscle cells; ECM, extracellular matrix; IL-1, interleukin-1; MDA, Malondialdehyde; T cells, T lymphocyte; TNF, tumor necrosis factor.

Ferroptosis, as a mode of cell death, plays an important role in COPD, asthma, lung injury, lung cancer, pulmonary fibrosis and lung infection. Therefore, it seems important to investigate the relationship between lung disease and ferroptosis to provide new ideas for clinical treatment (**Figure 5**).

### 6.1 COPD

COPD is an incompletely reversible systemic disease with airflow limitation. The pathological changes are mainly airway remodeling and/or abnormal alveolar wall elasticity. Pulmonary function test is the main objective index, which is manifested as decreased forced expiratory volume in 1 s (FEV1) (77, 78). COPD occurs mainly due to hand environment and genetic influences, and continuous exposure to cigarette smoke is one of the important environmental factors leading to COPD and the most common (79).Tobacco smoke induces inflammatory factor infiltration through ER stress, causing hypoxia in small airways and alveoli, accelerating the progression of COPD and inducing ferroptosis in lung epithelial cells (80). As macrophages accumulate causing accumulation of the inflammatory factor LTB4, ACSL4 expression is upregulated, thereby inducing ferroptosis in lung epithelial cells (81). In addition, the stimulation of herb smoke caused the accumulation of unstable iron and the enhancement of lipid peroxidation, again indicating that ferroptosis is closely related to COPD (82). The above mechanisms were mainly triggered by NCOA4-mediated iron autophagy and were not significantly associated with the GPX4 defense pathway (83). NCOA4 plays an important role in the pathogenesis of emphysema in COPD by polarizing M2 macrophages and inducing the secretion of inflammatory cells in bronchial epithelial cells (84).

The mechanism of iron responsive element binding protein 2 (IREB2) susceptibility to COPD is different from that of smoking. Overexpression of IREB2 indirectly leads to a significant decrease in lung compliance and total lung capacity, while it can cause inflammatory infiltration to increase IL-6 expression, induce hepcidin to regulate iron homeostasis, and induce FEV1 decline in COPD mice (85, 86). In parallel, elevated levels of IREB2 lead to accumulation of labile iron pools (LIPs) and lipid peroxidation, triggering iron overload in lung epithelial cells. Ferroptosis of airway epithelial cells and alveolar epithelial cells induces airway remodeling and emphysema, respectively, thereby causing COPD (87). Appropriate iron supplementation delays the onset and progression of COPD.

DNA dioxygenase 10-11 translocation 2 (TET2) is an important demethylase that regulates cigarette induced lipid peroxidation by demethylating GPX4, thereby reducing ferroptosis in COPD airway epithelial cells (80). The combination of the methylation inhibitor 5 ′ -aza-2 ′ -deoxycytidine (5-aza) and the antioxidant n -acetylcysteine (NAC) was strongly resistant to the production of inflammatory mediators induced by cigarettes in COPD (23).

In conclusion, the role of pulmonary iron regulation as well as iron metabolism in COPD remains to be investigated.Targeted iron therapy and lipid-specific anti-oxidation may be a strategy for the treatment of COPD and needs to be more reliably validated.

### 6.2 Asthma

Asthma is a chronic inflammatory respiratory disease characterized by airway altitude sickness and reversible airflow limitation (88). The main clinical symptoms are recurrent cough, sputum, wheezing, etc., which seriously threaten human health and occur frequently in children and the elderly. Airway inflammation and airway altitude sickness (AHR) are the core link in the pathological changes and recurrent attacks of asthma (89). Asthma is a chronic airway inflammatory disease that is closely associated with inflammatory cell infiltration (lymphocytes, mast cells, eosinophils, neutrophils), type II cytokine secretion (IL-4, IL-13, IL-5), and airway epithelial cell damage (90, 91).Oxidative stress response is closely associated with inflammation and AHR (92). Multiple markers of lipid peroxidation have been identified in patients with asthma, further suggesting that the pathophysiology of asthma is associated with ferroptosis (93). Correlative studies have shown that alveolar epithelial cell iron content is positively correlated with the progression of asthma. Substantial iron deposition is found in acellular cells of asthmatic patients causing airway inflammation and oxidative stress, thereby inducing ferroptosis (94). Among them, allergen exposure increases ROS production, breaks oxidative balance, accelerates oxidative stress progression, and is also responsible for aggravating asthma symptoms (95).

The ferroptosis defense pathway associated with asthma is mainly the Nrf2 pathway, which plays a role in resisting the progression of asthma course through anti-inflammatory mechanisms (96). Combined with ARE, Nrf2 inhibits the expression of type II cytokines in airway epithelial cells, inhibits oxidative stress, and slows the symptoms and signs of asthma (97). Nrf2-Keap1 signaling not only resists oxidative effects in asthma, but also promotes the expression of systemic Xc- and GPX4 factors, regulates SLC7A11 activity, reduces ROS production, and ultimately inhibits ferroptosis (98). Recent studies have found that licorice can activate the activity of Nrf2 and play a protective role in airway altitude sickness, providing new ideas for the clinical treatment of asthma (99).

15LO1, an abundant lipid peroxidase in airway epithelial cells (HAECs) of asthmatic patients, promotes the development of asthma, and its expression level is positively correlated with the severity of asthma (100). Type II cytokines interact with 15LO1 to promote inflammatory cell infiltration to induce inflammatory responses (101). 15LO1 binds to phosphatidylethanolamine-binding protein 1 (PEBPl) under cytokine stimulation, activates lipid peroxidation, and promotes ferroptosis in airway epithelial cells of asthmatic patients (102). It can be seen that specific inhibitors of 15LO1 are expected to provide new strategies for the treatment of steroid-resistant asthma.

### 6.3 Lung injury

#### 6.3.1 Acute lung injury

ALI is mainly characterized by alveolar epithelial cell damage, pulmonary interstitial edema, and neutrophil infiltration (103). Clinically, the main symptoms are decreased lung compliance and bilateral pulmonary inflammatory infiltrates in hypoxemia (104). Ferroptosis is the main driver of ischemia-reperfusion injury (I/R) and is closely related to the pathogenesis of lipopolysaccharide (LPS) -induced septic ALI, and can aggravate further tissue and organ damage (73, 105). Activation of NRF2 enhances resistance to lipid peroxidation induced lung injury by ferroptosis factors (GPX4, SlC7A11) in murine models of ALI, thereby protecting alveolar epithelial cells from ferroptosis (106, 107). In addition, the metabolites obacunone and itaconate significantly reduced lung injury by inducing activation of the NRF2 pathway (108, 109). By observing the ALI model, we could find that MDA expression increased, GSH and GPX4 expression decreased, and mitochondrial morphology changed in order to assess the degree of lung damage caused by ferroptosis at different I/R durations (110, 111).

In addition, the cells involved in ALI pathology and inflammatory response were mainly mast cells (MCs) and polymorphonuclear neutrophils (PMN) (112). MC is involved in the progression of ALI after autologous liver transplantation by regulating PMN apoptosis (113). At the same time, Nrf2 factor plays a protective role against sepsis-induced lung injury by activating antioxidant enzyme responses (108). However, the clinical treatment of ALI is still an exploratory stage at home and abroad. Currently, an increasing number of studies have shown that lipid peroxidation, which is a key cause of ferroptosis, plays an important role in ALI severity (114). Therefore, ferroptosis is closely related to ALI and may become an important therapeutic target for ALI (115).

#### 6.3.2 Radiation-induced lung injury

Radiotherapy is one of the important methods to treat lung tumors, and RILI (radiation pneumonitis and radiation pulmonary fibrosis) caused by radiotherapy is a common complication (116).

Radiation promotes the development of RILI, mainly promotes the infiltration of inflammatory cells to secrete various chemokines. ROS produced by radiation may be the original trigger for inducing ferroptosis in RILI, and the Keap1-NRF2 pathway has a protective effect against radiation-induced ferroptosis in alveolar epithelial cells (117). In a mouse model of RILI, inhibition of expression of the key factor GPX4 induced ROS accumulation leading to lipid peroxidation, further suggesting that ferroptosis RILI plays an important role. In addition, it has been found that the cascade of multiple cytokines is also involved in the process of ferroptosis in radiation-induced ALI. Transcription growth factor β1 (TGF-β1) and ROS synergize to promote ferroptosis in radiation-induced ALI and jointly aggravate lung injury, while Nrf2 slows radiation-induced ALI and the development of ferroptosis by reducing the expression of TGF-β1 and inhibiting iron ion absorption (118). ferroptosis has now been identified as playing an important role in radiation-induced ALI. Ferroptosis inhibitors may be an effective treatment for radiation-induced ALI, providing new insights into reducing ROS damage, preventing and treating radiation-induced lung injury. Ferroptosis inducers synergized with radiotherapy by enhancing cytoplasmic lipid peroxidation without increasing DNA damage or caspase activation, whereas ferroptosis inhibitors inhibited radiation-induced RILI and ferroptosis by inhibiting lipid peroxidation and enhancing GPX4 expression (119, 120).

### 6.4 Lung cancer

Lung cancer is one of the most common malignant tumors worldwide, with a very high morbidity and mortality (121), and its pathological types mainly include non-small cell lung cancer (NSCLC) and small cell lung cancer (SCLC) (122, 123). NSCLC is the most common type of lung cancer and is divided into lung adenocarcinoma (LUAD), lung squamous cell carcinoma (LUSC), and large cell carcinoma. In recent years, the incidence of LUAD has increased.

Cysteine desulfurase (NFS1) acts on lung tissue in a hyperoxic environment and there are amplified regions of the LUAD genome that are highly expressed. Simple inhibition of NFS1 activity does not slow LUAD growth and requires co-activation of the iron starvation response in cooperation with inhibition of glutathione organisms to trigger ferroptosis *in vitro*. Iron metabolism imbalance is closely related to the occurrence and development of lung cancer. USP35 combined with transportin (FPN) synergistically stabilized serum ferritin levels and inhibited ferroptosis to delay lung cancer cell growth (124).

SLC7A11 is centrally expressed in LUSC and is involved in body regulation (125). SLC7A11 expression increased in response to stimulation with the transcription factor SOX2, rendering lung cancer cells more resistant to upper iron (126). The expression of SLC7A11 and SOX2 was positively correlated in LSCC, and SOX2 plays an important role in the squamous cell fate of cells of different origins. In addition, RBMS1 acts as an RNA-binding protein and promotes ferroptosis by binding to the 3 ′ UTR region of SLC7A11 thereby promoting its translation and inhibiting SLC7A11-mediated cystine uptake (127).

SCLC is characterized by poor malignancy and prognosis and early metastasis (128). SCLC accounts for 15% of lung cancers and is associated with smoking, including neuroendocrine (NE) and non-neurosecretory (Non-NE) types (129). NE-SCLC are more susceptible to ferroptosis through isoform-specific lipidome remodeling, breaking oxidative balance (125). Chemotherapy resistance in Non-NE types is well-known because of peroxidation of specific membrane lipids of ACSL4 and LPCAT3, which induce ferroptosis. Combined treatment with ferroptosis mechanisms improves survival in lung cancer patients by inhibiting single-pathway targeting observed isoform plasticity.

### 6.5 Fibrotic lung diseases

Pulmonary fibrosis is a lung disease in which fibroblasts proliferate, a large amount of extracellular matrix accumulates leading to scarring, accompanied by inflammatory cell infiltration and destruction of alveolar wall structure (130, 131). At present, the pathogenesis and mechanism of pulmonary fibrosis are not clear, and there is a lack of effective treatment, and the prognosis of this patient is often poor. While the finding of ferroptosis is closely related to pulmonary fibrosis, it plays a key role in the pathogenesis of pulmonary fibrosis with iron overload, ROS accumulation, lipid peroxidation, and inhibition of GPX4 activity. When the level of iron in the body increases, it promotes the transformation of fibroblasts into myofibroblasts and accelerates the development of pulmonary fibrosis (132).

Ferroptosis is involved in the pathogenesis of pulmonary fibrosis, and the main triggers are ROS accumulation and glutathione depletion. The imbalance of antioxidant system is the key factor causing ROS accumulation and the occurrence and development of early pulmonary fibrosis. Nrf2-ARE, as an important pathway of ferroptosis, reduces the expression of free iron and smooth muscle actin by up-regulating the expression of HO-1, reduces collagen fiber synthesis, and finally inhibits ferroptosis-related pulmonary fibrosis (133). Type II alveolar epithelial cells (ATll) are critical cells for maintaining alveolar structure and function. The cell membrane contains a large number of polyunsaturated fatty acids and abundant mitochondrial content, and has a high susceptibility to ferroptosis (134). In bleomycin(BLM)-induced pulmonary fibrosis mouse specimens, ATII was found to contain a large number of iron ions, accompanied by pathological changes of fibrosis such as collagen deposition (135–137). Also, Fcn B secreted by exosomes from blm-induced alveolar macrophages promotes lung injury and fibrosis via ferroptosis in a blm-induced mouse model (137).

Liproxstatin-1, an ferroptosis inhibitor, delayed fibroblast differentiation into myofibroblasts and reduced pulmonary fibrosis by limiting collagen deposition and decreasing GPX4 expression (138, 139). Paraquat (PQ) is a highly toxic pesticide that causes diffuse fibrosis of the lungs.The toxic mechanism of PQ is mainly ROS imbalance leading to lipid peroxidation, mitochondrial damage, resulting in cellular ferroptosis (140). Recent studies have found that fine particulate matter (PM2.5) degrades heme-containing proteins through HO-1 and releases iron in fibrotic cells, resulting in mitochondrial ROS production, induced ferroptosis and aggravated pulmonary fibrosis (141). However, ferroptosis inhibitors such as desferrioxamine and Er-1 can play a key role in the treatment of pulmonary fibrosis induced by PQ and PM2.5.

### 6.6 Pulmonary infection

#### 6.6.1 Tuberculosis

Tuberculosis (TB) is caused by Mycobacterium tuberculosis (Mtb) infection causing chronic infectious diseases that affect human life and health. TB is the main cause of death caused by a single source of infection (142). With the incidence of multi-drug resistant tuberculosis increasing year by year, the prevention and treatment situation is very severe, and anti-tuberculosis treatment is of great significance in clinical practice. Mtb is highly contagious, and when inhaled into the body, it activates macrophages in the alveoli to produce an adaptive immune response, thereby eliminating Mycobacterium tuberculosis. At the same time, Mtb can also evade macrophage killing by inducing macrophage necrosis through negative regulation. In recent years, increasing evidence suggests that ferroptosis is significantly associated with pathogenicity and dissemination of Mtb. In mice acutely infected with Mtb, alveolar macrophage necrosis was significantly associated with a phenotype of ferroptosis, mainly characterized by decreased Gpx4 expression, increased lipid peroxidation, and mitochondrial hyperoxidation (143). Protein tyrosine phosphatase A (PtpA) secreted by Mtb interacts with host RanGDP to enter the nucleus and promotes arginine methyltransferase 6 (PRMT6) -mediated methylation of H3R2me2a on the GPX4 promoter, resulting in decreased GPX4 expression and ferroptosis induction in host cells and promoting Mtb pathogenicityand dissemination (144). These results suggest that gpx4-dependent iron cell apoptosis may be targeted by blocking the Mtb ptpa- host PRMT6 interface, providing a new therapeutic strategy for the treatment of tuberculosis (145).

Subsequently, GPX4 knockout mice were found to aggravate TB infection, while overexpression of GPX4 significantly reduced bacterial load and risk of infection. Meanwhile, Fer-1 could reduce lipid peroxidation and inhibit cellular ferroptosis in Mtb-infected macrophages (146). In summary, ferroptosis is closely related to the occurrence and development of Mtb infection, and inhibition of ferroptosis can inhibit Mtb infection and pulmonary inflammatory response.

#### 6.6.2 Coronavirus disease 2019

It is a pulmonary infectious disease caused by SARS-CoV-2 virus infection causing severe acute respiratory distress (147). SARS-CoV-2 targets various systemic functions of the body, with the lungs and throat of the respiratory system as the main targets (148). Abnormal lipid expression in pneumocytes was found to result in increased pneumocyte apoptosis and ferroptosis in mice infected with SARS-CoV-2 virus (149). Reactive breakdown products of lipid peroxides were observed in a case of severe COVID-19 infection with myocarditis (150). In addition, SARS-CoV-2 inhibited GPX4 mRNA expression to induce apoptosis by attacking lung macrophages and monocytes (151). Related studies showed that expression levels of major signaling factors for ferroptosis, including GPX4 and SLC7A11, were significantly upregulated in sera from patients with novel coronavirus infection (152, 153).

Recent studies have found that SARS-CoV-2 ORF3a can increase the sensitivity of cells to iron ions through the Keap1-NRF2 axis on the one hand, accelerate the degradation of NRF2 by Keap1 on the other hand, weaken the resistance of cells to oxidative stress, and induce ferroptosis in cells on the other hand (154). In addition, vitamin K reduces the level of reactive oxygen species by regulating the expression of antioxidant enzymes and can also prevent ferroptosis by reducing the inflammatory response (155).

In summary, ferroptosis plays a diversified role in COVID-19, and understanding the signaling mechanism of ferroptosis during SARS-CoV-2 virus infection will help to advance the clinical treatment and drug research and development of the disease, and targeting iron-tropic organics seems to be a potential novel therapeutic strategy for COVID-19.

#### 6.6.3 Pseudomonas aeruginosa

Respiratory diseases are inextricably linked to respiratory microbiota infections, and Pseudomonas aeruginosa (PA) is one of the most common pathogenic groups (156). PA is the main species of nosocomial infection, which can be found in most patients with long-term mechanical ventilation in intensive care units and is also an important opportunistic pathogen causing acute lung injury and acute respiratory distress syndrome (157). PA contains secretory vesicles that catalyze the host PUFA reaction by 15LOX, thereby making virulence factors to induce ferroptosis (158). Although PA uses LOX to participate in the ferroptosis process, 15LOX has a significant lack of lipid substrates (159). Also, it has been shown that PA decreases the effects of host GPx4 to induce lipid peroxidation by activating lysosomal chaperone-mediated autophagy (160). Using a macrophage infection model, P. aeruginosa RNase E variants cause host infection damage by increasing host cell siderophore production and iron cell apoptosis (161). In summary, PA is closely related to ferroptosis, while 15LOX-induced ferroptosis progression serves as a therapeutic target, providing new therapeutic ideas for non-antibiotic treatment of PA-induced airway infections.

## 7 Targeting ferroptosis in lung disease

COPD is associated with iron imbalance, and treatment to correct disorders of body iron metabolism may be helpful in the treatment of the disease. In a mouse model exposed to cigarettes, regarding the progression of resistance to COPD, with the exception of GPx4 knockdown, desferrioxamine and ferristatin-1 are a possible target for the treatment of ferroptosis-induced COPD (82). At the same time, chelators, iron supplementation, or low-iron diets are current methods to correct iron levels and avoid COPD damage to the body.

Asthma is characterized by recurrent and difficult to cure as the main clinical features. Relevant clinical and serial experiments have shown that acupuncture has a regulatory effect on mucosal and cellular immunity in patients with allergic asthma and may be an adjuvant method for the treatment of asthma (162, 163). In the mouse asthma model treated with acupuncture, the expression of ferroptosis regulator MDA was down-regulated and GSH was up-regulated, further elaborating that the effect of acupuncture on asthma is associated with the regulation of ferroptosis.However, the regulatory mechanisms involved need to be investigated.

Studies have shown that iron sag is an effective mechanism to induce ALI, and inhibition of iron sag provides a more reliable means to prevent and treat ALI induced by i/R or lps. Liprostatin-1 and Ferrostatin-1 were able to ameliorate lung histopathological damage, pulmonary edema alterations, and lipid peroxidation progression in I/R mice (107, 110).

Ferroptosis has become an effective therapeutic target for lung cancer, especially for lung cancer types with drug resistance (164, 165). Platinum inhibits iron cell apoptosis by high depletion of GSH through activation of the Wnt/NR2F2/GPX4 pathway.GPX4 inhibitors have been found to enhance the anticancer effects of Platinum providing new therapeutic ideas for lung cancer patients (166). In addition, nanotechnology of tumor *in situ* iron mineralization provides a new scheme for the early diagnosis of lung cancer, using Prussian blue/calcium peroxide nanocomposite technology to induce iron mineralization in lung cancer cells, while causing oxidative stress to induce apoptosis and ferroptosis, and inhibiting the malignant growth of tumor cells (167). Recent studies have found that self-assembled ph sensitive superparamagnetic iron oxide nanoclusters (SPIONCs) technology kills lung tumor cells by participating in the Fenton response and inducing ferroptosis in an acidic environment through radiation therapy and iron ion release (168). Immunotherapy is currently one of the effective methods for anti-lung cancer tumor therapy, in which immune checkpoint inhibitors (ICIs) mainly exert anti-tumor effects by activating T cells, and currently approved ICIs have drugs targeting CTLA4, PD-1 and PD-L1 (169). Activated CD8^+^ T cells release interferon-gamma, down-regulate the expression of SLC7A11 and SLC3A2, and inhibit the uptake of cystine by lung cancer cells to achieve anti-tumor effects (170). In addition, some nanoparticles induce ferroptosis to achieve inhibition of lung cancer tumors, of which SRFFe IITA (SFT) and zero-valent iron nanoparticles (ZVI-NP) are effectively combined with ferroptosis by photodynamic therapy and immunostimulation to treat lung tumors (171, 172).

In the physiological and pathological changes of pulmonary fibrosis, liproxstatin-1 can activate the Nrf2 pathway to downregulate transforming growth factor β1 (TGF-β1) and delay the progression of pulmonary fibrosis (118). Substantial iron deposition was found in alveolar epithelial cells of lung fibrosis samples, whereas deferoxamine (DFO) prevented lung fibrosis progression and ferroptosis by stabilizing iron metabolism in the lung (136). In addition, SODARA290-HBc, a newly constructed bioengineering nanoreactor, protects alveolar epithelial cells from radiation and iron poisoning by inhibiting oxidative stress, inflammation, and regulating the phenotype of infiltrating macrophages in the RILI mouse model (173).

Iron metabolism disorders are inextricably linked to the development of pulmonary tuberculosis, and biomarker levels of iron ions provide new ideas for the diagnosis of pulmonary tuberculosis (174). Isoniazid (INH), as an anti-tuberculosis drug, damages the liver and induces lipid peroxidation leading to apoptosis of liver cells through glutathione depletion (175). Thus, anti-ferroptosis appears to be an effective target for treating TB or delaying TB progression.

SARS-CoV-2 virus is a pathogenic agent of novel coronavirus infection and is closely associated with the development of ferroptosis. Studies have shown that iron-sulfur cofactor is a cofactor of SARS-CoV-2 virus and a therapeutic target of COVID-19 (176). Two candidates, DFO and imatinib, were identified to be effective in blocking SARS-CoV-2 infection and infection-related ferroptosis in a mouse model of sinoatrial node-like pacemaker cell dysfunction infected with novel coronavirus (177).

## 8 Conclusion

For ferroptosis, as research progresses, the documented related signaling pathways between induction and inhibition mechanisms, as well as the regulatory pathways of ferroptosis, are explored. This paper also reviews the relationship between ferroptosis and respiratory diseases. However, despite increasing evidence from animal experimentation demonstrating the effectiveness of targeted ferroptosis therapy in lung disease, questions remain regarding its clinical role.

SLC7A11 and GPX4 are important regulators of ferroptosis, and multiple ferroptosis inducers exert anti-ferroptosis effects in alveolar cells through them. Specifically, high expression of SLC7A11 and GPX4 is a potential target for the treatment of lung diseases. However, expression levels of SLC7A11 and GPX4 differ in a variety of respiratory diseases. Therefore, it becomes particularly important to screen key genes that provide relevant evidence for targeted therapy for clinical ferroptosis. In addition, an increasing number of studies have shown that ferroptosis, as an adjuvant clinical treatment option, will become a new target for the treatment of various lung diseases that are currently incurable.

## Author contributions

FZ: Writing – original draft. YX: Writing – original draft. QM: Writing – original draft. EG: Writing – review & editing. XZ: Writing – review & editing.

## References

[1] Yan R Xie E Li Y Li J Zhang Y Chi X. The structure of erastin-bound xct-4f2hc complex reveals molecular mechanisms underlying erastin-induced ferroptosis. Cell Res. (2022) 32:687–90. doi: 10.1038/s41422-022-00642-w PMC9253326 35352032

[2] Chen H Qi Q Wu N Wang Y Feng Q Jin R. Aspirin promotes rsl3-induced ferroptosis by suppressing mtor/srebp-1/scd1-mediated lipogenesis in pik3ca-mutant colorectal cancer. Redox Biol. (2022) 55:102426. doi: 10.1016/j.redox.2022.102426 35963119 PMC9389304

[3] Dixon SJ Lemberg Km Fau - Lamprecht MR Lamprecht Mr Fau - Skouta R Skouta R Fau - Zaitsev EM Zaitsev Em Fau - Gleason CE Gleason Ce Fau - Patel DN. Ferroptosis: an iron-dependent form of nonapoptotic cell death. Cell. (2012) 149(5):1060–72. doi: 10.1016/j.cell.2012.03.042 22632970 PMC3367386

[4] Lin Z Liu J Long F Kang R Kroemer G Tang D. The lipid flippase slc47a1 blocks metabolic vulnerability to ferroptosis. Nat Commun. (2022) 13:7965. doi: 10.1038/s41467-022-35707-2 36575162 PMC9794750

[5] Gao M Yi J Zhu J Minikes AM Monian P Thompson CB. Role of mitochondria in ferroptosis. Mol Cell. (2019) 73:354–63.e3. doi: 10.1016/j.molcel.2018.10.042 30581146 PMC6338496

[6] Li C Zhang Y Liu J Kang R Klionsky DJ Tang D. Mitochondrial DNA stress triggers autophagy-dependent ferroptotic death. Autophagy. (2021) 17:948–60. doi: 10.1080/15548627.2020.1739447 PMC8078708 32186434

[7] Lee H Zandkarimi F Zhang Y Meena JK Kim J Zhuang L. Energy-stress-mediated ampk activation inhibits ferroptosis. Nat Cell Biol. (2020) 22:225–34. doi: 10.1038/s41556-020-0461-8 PMC7008777 32029897

[8] Sang M Luo R Bai Y Dou J Zhang Z Liu F. Mitochondrial membrane anchored photosensitive nano-device for lipid hydroperoxides burst and inducing ferroptosis to surmount therapy-resistant cancer. Theranostics. (2019) 9:6209–23. doi: 10.7150/thno.36283 PMC6735518 31534546

[9] Man WH de Steenhuijsen Piters WA Bogaert D. The microbiota of the respiratory tract: gatekeeper to respiratory health. Nat Rev Microbiol. (2017) 15:259–70. doi: 10.1038/nrmicro.2017.14 PMC7097736 28316330

[10] Wu X Li Y Zhang S Zhou X. Ferroptosis as a novel therapeutic target for cardiovascular disease. Theranostics. (2021) 11:3052–9. doi: 10.7150/thno.54113 PMC7847684 33537073

[11] Chen X Kang R Kroemer G Tang D. Broadening horizons: the role of ferroptosis in cancer. Nat Rev Clin Oncol. (2021) 18:280–96. doi: 10.1038/s41571-020-00462-0 33514910

[12] Friedmann Angeli JP Schneider M Proneth B Tyurina YY Tyurin VA Hammond VJ. Inactivation of the ferroptosis regulator gpx4 triggers acute renal failure in mice. Nat Cell Biol. (2014) 16:1180–91. doi: 10.1038/ncb3064 PMC4894846 25402683

[13] Yuan T Zhang H Chen D Chen Y Lyu Y Fang L. Puerarin protects pulmonary arteries from hypoxic injury through the bmprii and pparγ Signaling pathways in endothelial cells. Pharmacol Rep. (2019) 71:855–61. doi: 10.1016/j.pharep.2019.05.002 31408784

[14] Holt PG Strickland DH Wikström ME Jahnsen FL. Regulation of immunological homeostasis in the respiratory tract. Nat Rev Immunol. (2008) 8:142–52. doi: 10.1038/nri2236 18204469

[15] Liu X Chen Z. The pathophysiological role of mitochondrial oxidative stress in lung diseases. J Transl Med. (2017) 15:207. doi: 10.1186/s12967-017-1306-5 29029603 PMC5640915

[16] Yang L Cao LM Zhang XJ Chu B. Targeting ferroptosis as a vulnerability in pulmonary diseases. Cell Death Dis. (2022) 13:649. doi: 10.1038/s41419-022-05070-7 35882850 PMC9315842

[17] Gao J Wang Q Tang YD Zhai J Hu W Zheng C. When ferroptosis meets pathogenic infections. Trends Microbiol. (2023) 31:468–79. doi: 10.1016/j.tim.2022.11.006 36496309

[18] Anderson GJ Frazer DM. Current understanding of iron homeostasis. Am J Clin Nutr. (2017) 106:1559s–66s. doi: 10.3945/ajcn.117.155804 PMC5701707 29070551

[19] Galy B Conrad M Muckenthaler M. Mechanisms controlling cellular and systemic iron homeostasis. Nat Rev Mol Cell Biol. (2023) 25(2):133–55. doi: 10.1038/s41580-023-00648-1 37783783

[20] Wilkinson HN Upson SE Banyard KL Knight R Mace KA Hardman MJ. Reduced iron in diabetic wounds: an oxidative stress-dependent role for steap3 in extracellular matrix deposition and remodeling. J Invest Dermatol. (2019) 139:2368–77.e7. doi: 10.1016/j.jid.2019.05.014 31176711

[21] Montalbetti N Simonin A Kovacs G Hediger MA. Mammalian iron transporters: families slc11 and slc40. Mol Aspects Med. (2013) 34:270–87. doi: 10.1016/j.mam.2013.01.002 23506870

[22] Luo P Liu D Zhang Q Yang F Wong YK Xia F. Celastrol induces ferroptosis in activated hscs to ameliorate hepatic fibrosis via targeting peroxiredoxins and ho-1. Acta Pharm Sin B. (2022) 12:2300–14. doi: 10.1016/j.apsb.2021.12.007 PMC9136576 35646542

[23] Li Y Yang Y Guo T Weng C Yang Y Wang Z. Heme oxygenase-1 determines the cell fate of ferroptotic death of alveolar macrophages in copd. Front Immunol. (2023) 14:1162087. doi: 10.3389/fimmu.2023.1162087 37215140 PMC10196003

[24] Hentze MW Muckenthaler MU Galy B Camaschella C. Two to tango: regulation of mammalian iron metabolism. Cell. (2010) 142:24–38. doi: 10.1016/j.cell.2010.06.028 20603012

[25] Muckenthaler MU Galy B Hentze MW. Systemic iron homeostasis and the iron-responsive element/iron-regulatory protein (Ire/irp) regulatory network. Annu Rev Nutr. (2008) 28:197–213. doi: 10.1146/annurev.nutr.28.061807.155521 18489257

[26] Conrad M Pratt DA. The chemical basis of ferroptosis. Nat Chem Biol. (2019) 15:1137–47. doi: 10.1038/s41589-019-0408-1 31740834

[27] Liang D Minikes AM Jiang X. Ferroptosis at the intersection of lipid metabolism and cellular signaling. Mol Cell. (2022) 82:2215–27. doi: 10.1016/j.molcel.2022.03.022 PMC9233073 35390277

[28] He J Peng H Wang M Liu Y Guo X Wang B. Isoliquiritigenin inhibits tgf-B1-induced fibrogenesis through activating autophagy via pi3k/akt/mtor pathway in mrc-5 cells. Acta Biochim Biophys Sin (Shanghai). (2020) 52:810–20. doi: 10.1093/abbs/gmaa067 32638014

[29] Dixon SJ Winter GE Musavi LS Lee ED Snijder B Rebsamen M. Human haploid cell genetics reveals roles for lipid metabolism genes in nonapoptotic cell death. ACS Chem Biol. (2015) 10:1604–9. doi: 10.1021/acschembio.5b00245 PMC4509420 25965523

[30] Yin H Xu L Porter NA. Free radical lipid peroxidation: mechanisms and analysis. Chem Rev. (2011) 111:5944–72. doi: 10.1021/cr200084z 21861450

[31] Su LJ Zhang JH Gomez H Murugan R Hong X Xu D. Reactive oxygen species-induced lipid peroxidation in apoptosis, autophagy, and ferroptosis. Oxid Med Cell Longev. (2019) 2019:5080843. doi: 10.1155/2019/5080843 31737171 PMC6815535

[32] Ursini F Maiorino M. Lipid peroxidation and ferroptosis: the role of gsh and gpx4. Free Radic Biol Med. (2020) 152:175–85. doi: 10.1016/j.freeradbiomed.2020.02.027 32165281

[33] Hadian K Stockwell BR. Snapshot: ferroptosis. Cell. (2020) 181:1188–.e1. doi: 10.1016/j.cell.2020.04.039 PMC8157339 32470402

[34] Cai W Liu L Shi X Liu Y Wang J Fang X. Alox15/15-hpete aggravates myocardial ischemia-reperfusion injury by promoting cardiomyocyte ferroptosis. Circulation. (2023) 147:1444–60. doi: 10.1161/circulationaha.122.060257 36987924

[35] Shah R Shchepinov MS Pratt DA. Resolving the role of lipoxygenases in the initiation and execution of ferroptosis. ACS Cent Sci. (2018) 4:387–96. doi: 10.1021/acscentsci.7b00589 PMC5879472 29632885

[36] Upadhyayula PS Higgins DM Mela A Banu M Dovas A Zandkarimi F. Dietary restriction of cysteine and methionine sensitizes gliomas to ferroptosis and induces alterations in energetic metabolism. Nat Commun. (2023) 14:1187. doi: 10.1038/s41467-023-36630-w 36864031 PMC9981683

[37] Liu Y Liu S Tomar A Yen FS Unlu G Ropek N. Autoregulatory control of mitochondrial glutathione homeostasis. Science. (2023) 382:820–8. doi: 10.1126/science.adf4154 PMC11170550 37917749

[38] Dangol S Chen Y Hwang BK Jwa NS. Iron- and reactive oxygen species-dependent ferroptotic cell death in rice-magnaporthe oryzae interactions. Plant Cell. (2019) 31:189–209. doi: 10.1105/tpc.18.00535 30563847 PMC6391706

[39] Wang L Liu Y Du T Yang H Lei L Guo M. Atf3 promotes erastin-induced ferroptosis by suppressing system xc(). Cell Death Differ. (2020) 27:662–75. doi: 10.1038/s41418-019-0380-z PMC7206049 31273299

[40] Dixon SJ Stockwell BR. The role of iron and reactive oxygen species in cell death. Nat Chem Biol. (2014) 10:9–17. doi: 10.1038/nchembio.1416 24346035

[41] Steinberg GR Hardie DG. New insights into activation and function of the ampk. Nat Rev Mol Cell Biol. (2023) 24:255–72. doi: 10.1038/s41580-022-00547-x 36316383

[42] Li C Dong X Du W Shi X Chen K Zhang W. Lkb1-ampk axis negatively regulates ferroptosis by inhibiting fatty acid synthesis. Signal Transduct Target Ther. (2020) 5:187. doi: 10.1038/s41392-020-00297-2 32883948 PMC7471309

[43] Zhong S Chen W Wang B Gao C Liu X Song Y. Energy stress modulation of ampk/foxo3 signaling inhibits mitochondria-associated ferroptosis. Redox Biol. (2023) 63:102760. doi: 10.1016/j.redox.2023.102760 37267686 PMC10244700

[44] Xiao Y Xie X Chen Z Yin G Kong W Zhou J. Advances in the roles of atf4 in osteoporosis. BioMed Pharmacother. (2023) 169:115864. doi: 10.1016/j.biopha.2023.115864 37948991

[45] Wang N Zeng GZ Yin JL Bian ZX. Artesunate activates the atf4-chop-chac1 pathway and affects ferroptosis in burkitt’s lymphoma. Biochem Biophys Res Commun. (2019) 519:533–9. doi: 10.1016/j.bbrc.2019.09.023 31537387

[46] Fan X Dong T Yan K Ci X Peng L. Pm2.5 increases susceptibility to acute exacerbation of copd via nox4/nrf2 redox imbalance-mediated mitophagy. Redox Biol. (2023) 59:102587. doi: 10.1016/j.redox.2022.102587 36608590 PMC9813701

[47] Kuroda J Ago T Matsushima S Zhai P Schneider MD Sadoshima J. Nadph oxidase 4 (Nox4) is a major source of oxidative stress in the failing heart. Proc Natl Acad Sci USA. (2010) 107:15565–70. doi: 10.1073/pnas.1002178107 PMC2932625 20713697

[48] Yuan B Liu J Shi A Cao J Yu Y Zhu Y. Herc3 promotes yap/taz stability and tumorigenesis independently of its ubiquitin ligase activity. EMBO J. (2023) 42:e111549. doi: 10.15252/embj.2022111549 36598329 PMC9929636

[49] Zanconato F Cordenonsi M Piccolo S. Yap/taz at the roots of cancer. Cancer Cell. (2016) 29:783–803. doi: 10.1016/j.ccell.2016.05.005 27300434 PMC6186419

[50] Liu J Yang G Zhang H. Glyphosate-triggered hepatocyte ferroptosis via suppressing nrf2/gsh/gpx4 axis exacerbates hepatotoxicity. Sci Total Environ. (2023) 862:160839. doi: 10.1016/j.scitotenv.2022.160839 36521597

[51] Ingold I Berndt C Schmitt S Doll S Poschmann G Buday K. Selenium utilization by gpx4 is required to prevent hydroperoxide-induced ferroptosis. Cell. (2018) 172:409–22.e21. doi: 10.1016/j.cell.2017.11.048 29290465

[52] Brigelius-Flohé R Maiorino M. Glutathione peroxidases. Biochim Biophys Acta. (2013) 1830:3289–303. doi: 10.1016/j.bbagen.2012.11.020 23201771

[53] Chio IIC Tuveson DA. Ros in cancer: the burning question. Trends Mol Med. (2017) 23:411–29. doi: 10.1016/j.molmed.2017.03.004 PMC5462452 28427863

[54] Li W Liang L Liu S Yi H Zhou Y. Fsp1: A key regulator of ferroptosis. Trends Mol Med. (2023) 29:753–64. doi: 10.1016/j.molmed.2023.05.013 37357101

[55] Doll S Freitas FP Shah R Aldrovandi M da Silva MC Ingold I. Fsp1 is a glutathione-independent ferroptosis suppressor. Nature. (2019) 575:693–8. doi: 10.1038/s41586-019-1707-0 31634899

[56] Wang D Liang W Huo D Wang H Wang Y Cong C. Spy1 inhibits neuronal ferroptosis in amyotrophic lateral sclerosis by reducing lipid peroxidation through regulation of gch1 and tfr1. Cell Death Differ. (2023) 30:369–82. doi: 10.1038/s41418-022-01089-7 PMC9950139 36443440

[57] Kraft VAN Bezjian CT Pfeiffer S Ringelstetter L Müller C Zandkarimi F. Gtp cyclohydrolase 1/tetrahydrobiopterin counteract ferroptosis through lipid remodeling. ACS Cent Sci. (2020) 6:41–53. doi: 10.1021/acscentsci.9b01063 31989025 PMC6978838

[58] Soula M Weber RA Zilka O Alwaseem H La K Yen F. Metabolic determinants of cancer cell sensitivity to canonical ferroptosis inducers. Nat Chem Biol. (2020) 16:1351–60. doi: 10.1038/s41589-020-0613-y PMC8299533 32778843

[59] Mao C Liu X Zhang Y Lei G Yan Y Lee H. Dhodh-mediated ferroptosis defence is a targetable vulnerability in cancer. Nature. (2021) 593:586–90. doi: 10.1038/s41586-021-03539-7 PMC8895686 33981038

[60] Amos A Amos A Wu L Xia H. The warburg effect modulates dhodh role in ferroptosis: A review. Cell Commun Signal. (2023) 21:100. doi: 10.1186/s12964-022-01025-9 37147673 PMC10161480

[61] Ding Q Tang W Li X Ding Y Chen X Cao W. Mitochondrial-targeted brequinar liposome boosted mitochondrial-related ferroptosis for promoting checkpoint blockade immunotherapy in bladder cancer. J Control Release. (2023) 363:221–34. doi: 10.1016/j.jconrel.2023.09.024 37717657

[62] Mou Y Wang J Wu J He D Zhang C Duan C. Ferroptosis, a new form of cell death: opportunities and challenges in cancer. J Hematol Oncol. (2019) 12:34. doi: 10.1186/s13045-019-0720-y 30925886 PMC6441206

[63] Kerins MJ Ooi A. The roles of nrf2 in modulating cellular iron homeostasis. Antioxid Redox Signal. (2018) 29:1756–73. doi: 10.1089/ars.2017.7176 PMC6208163 28793787

[64] Anandhan A Dodson M Shakya A Chen J Liu P Wei Y. Nrf2 controls iron homeostasis and ferroptosis through herc2 and vamp8. Sci Adv. (2023) 9:eade9585. doi: 10.1126/sciadv.ade9585 36724221 PMC9891695

[65] Jiang C Ward NP Prieto-Farigua N Kang YP Thalakola A Teng M. A crispr screen identifies redox vulnerabilities for keap1/nrf2 mutant non-small cell lung cancer. Redox Biol. (2022) 54:102358. doi: 10.1016/j.redox.2022.102358 35667246 PMC9168196

[66] Costa I Barbosa DJ Benfeito S Silva V Chavarria D Borges F. Molecular mechanisms of ferroptosis and their involvement in brain diseases. Pharmacol Ther. (2023) 244:108373. doi: 10.1016/j.pharmthera.2023.108373 36894028

[67] Park SY Jeong KJ Poire A Zhang D Tsang YH Blucher AS. Irreversible her2 inhibitors overcome resistance to the rsl3 ferroptosis inducer in non-her2 amplified luminal breast cancer. Cell Death Dis. (2023) 14:532. doi: 10.1038/s41419-023-06042-1 37596261 PMC10439209

[68] Keldsen N Havsteen H Vergote I Bertelsen K Jakobsen A. Altretamine (Hexamethylmelamine) in the treatment of platinum-resistant ovarian cancer: A phase ii study. Gynecol Oncol. (2003) 88:118–22. doi: 10.1016/s0090-8258(02)00103-8 12586589

[69] Hassannia B Wiernicki B Ingold I Qu F Van Herck S Tyurina YY. Nano-targeted induction of dual ferroptotic mechanisms eradicates high-risk neuroblastoma. J Clin Invest. (2018) 128:3341–55. doi: 10.1172/jci99032 PMC6063467 29939160

[70] Xu Y Li K Zhao Y Zhou L Liu Y Zhao J. Role of ferroptosis in stroke. Cell Mol Neurobiol. (2023) 43:205–22. doi: 10.1007/s10571-022-01196-6 PMC11415219 35102454

[71] Liu Y Wang W Li Y Xiao Y Cheng J Jia J. The 5-lipoxygenase inhibitor zileuton confers neuroprotection against glutamate oxidative damage by inhibiting ferroptosis. Biol Pharm Bull. (2015) 38:1234–9. doi: 10.1248/bpb.b15-00048 26235588

[72] Codenotti S Poli M Asperti M Zizioli D Marampon F Fanzani A. Cell growth potential drives ferroptosis susceptibility in rhabdomyosarcoma and myoblast cell lines. J Cancer Res Clin Oncol. (2018) 144:1717–30. doi: 10.1007/s00432-018-2699-0 PMC11813460 29971532

[73] Li Y Feng D Wang Z Zhao Y Sun R Tian D. Ischemia-induced acsl4 activation contributes to ferroptosis-mediated tissue injury in intestinal ischemia/reperfusion. Cell Death Differ. (2019) 26:2284–99. doi: 10.1038/s41418-019-0299-4 PMC6889315 30737476

[74] Yang K Zeng L Zeng J Deng Y Wang S Xu H. Research progress in the molecular mechanism of ferroptosis in parkinson’s disease and regulation by natural plant products. Ageing Res Rev. (2023) 91:102063. doi: 10.1016/j.arr.2023.102063 37673132

[75] Chen Y Mi Y Zhang X Ma Q Song Y Zhang L. Dihydroartemisinin-induced unfolded protein response feedback attenuates ferroptosis via perk/atf4/hspa5 pathway in glioma cells. J Exp Clin Cancer Res. (2019) 38:402. doi: 10.1186/s13046-019-1413-7 31519193 PMC6743121

[76] Wang L Zhang Z Li M Wang F Jia Y Zhang F. P53-dependent induction of ferroptosis is required for artemether to alleviate carbon tetrachloride-induced liver fibrosis and hepatic stellate cell activation. IUBMB Life. (2019) 71:45–56. doi: 10.1002/iub.1895 30321484

[77] Fabbri LM Celli BR Agustí A Criner GJ Dransfield MT Divo M. Copd and multimorbidity: recognising and addressing a syndemic occurrence. Lancet Respir Med. (2023) 11:1020–34. doi: 10.1016/s2213-2600(23)00261-8 37696283

[78] Yang IA Jenkins CR Salvi SS. Chronic obstructive pulmonary disease in never-smokers: risk factors, pathogenesis, and implications for prevention and treatment. Lancet Respir Med. (2022) 10:497–511. doi: 10.1016/s2213-2600(21)00506-3 35427530

[79] Chen J Wang X Schmalen A Haines S Wolff M Ma H. Antiviral cd8(+) T-cell immune responses are impaired by cigarette smoke and in copd. Eur Respir J. (2023) 62(2):2201374. doi: 10.1183/13993003.01374-2022 37385655 PMC10397470

[80] Zeng Z Li T Liu X Ma Y Luo L Wang Z. DNA dioxygenases tet2 deficiency promotes cigarette smoke induced chronic obstructive pulmonary disease by inducing ferroptosis of lung epithelial cell. Redox Biol. (2023) 67:102916. doi: 10.1016/j.redox.2023.102916 37812881 PMC10579541

[81] Günes Günsel G Conlon TM Jeridi A Kim R Ertüz Z Lang NJ. The arginine methyltransferase prmt7 promotes extravasation of monocytes resulting in tissue injury in copd. Nat Commun. (2022) 13:1303. doi: 10.1038/s41467-022-28809-4 35288557 PMC8921220

[82] Yoshida M Minagawa S Araya J Sakamoto T Hara H Tsubouchi K. Involvement of cigarette smoke-induced epithelial cell ferroptosis in copd pathogenesis. Nat Commun. (2019) 10:3145. doi: 10.1038/s41467-019-10991-7 31316058 PMC6637122

[83] Cloonan SM Glass K Laucho-Contreras ME Bhashyam AR Cervo M Pabón MA. Mitochondrial iron chelation ameliorates cigarette smoke-induced bronchitis and emphysema in mice. Nat Med. (2016) 22:163–74. doi: 10.1038/nm.4021 PMC4742374 26752519

[84] Liu J Zhang Z Yang Y Di T Wu Y Bian T. Ncoa4-mediated ferroptosis in bronchial epithelial cells promotes macrophage M2 polarization in copd emphysema. Int J Chron Obstruct Pulmon Dis. (2022) 17:667–81. doi: 10.2147/copd.S354896 PMC8978690 35386390

[85] Hubeau C Kubera JE Masek-Hammerman K Williams CM. Interleukin-6 neutralization alleviates pulmonary inflammation in mice exposed to cigarette smoke and poly(I:C). Clin Sci (Lond). (2013) 125:483–93. doi: 10.1042/cs20130110 23738811

[86] Qiu C Li Y Li M Li M Liu X McSharry C. Anti-interleukin-33 inhibits cigarette smoke-induced lung inflammation in mice. Immunology. (2013) 138:76–82. doi: 10.1111/imm.12020 23078031 PMC3533703

[87] Xia H Wu Y Zhao J Cheng C Lin J Yang Y. N6-methyladenosine-modified circsav1 triggers ferroptosis in copd through recruiting ythdf1 to facilitate the translation of ireb2. Cell Death Differ. (2023) 30:1293–304. doi: 10.1038/s41418-023-01138-9 PMC10154389 36828914

[88] Wan R Srikaram P Guntupalli V Hu C Chen Q Gao P. Cellular senescence in asthma: from pathogenesis to therapeutic challenges. EBioMedicine. (2023) 94:104717. doi: 10.1016/j.ebiom.2023.104717 37442061 PMC10362295

[89] LeSuer WE Kienzl M Ochkur SI Schicho R Doyle AD Wright BL. Eosinophils promote effector functions of lung group 2 innate lymphoid cells in allergic airway inflammation in mice. J Allergy Clin Immunol. (2023) 152:469–85.e10. doi: 10.1016/j.jaci.2023.03.023 37028525 PMC10503660

[90] Papi A Brightling C Pedersen SE Reddel HK. Asthma. Lancet. (2018) 391:783–800. doi: 10.1016/s0140-6736(17)33311-1 29273246

[91] Peters MC Wenzel SE. Intersection of biology and therapeutics: type 2 targeted therapeutics for adult asthma. Lancet. (2020) 395:371–83. doi: 10.1016/s0140-6736(19)33005-3 PMC8522504 32007172

[92] Akel Bilgic H Kilic B Kockaya BD Sarac BE Kilic Suloglu A Kalayci O. Oxidative stress stimulation leads to cell-specific oxidant and antioxidant responses in airway resident and inflammatory cells. Life Sci. (2023) 315:121358. doi: 10.1016/j.lfs.2022.121358 36596408

[93] Sahiner UM Birben E Erzurum S Sackesen C Kalayci Ö. Oxidative stress in asthma: part of the puzzle. Pediatr Allergy Immunol. (2018) 29:789–800. doi: 10.1111/pai.12965 30069955

[94] Yu S Jia J Zheng J Zhou Y Jia D Wang J. Recent progress of ferroptosis in lung diseases. Front Cell Dev Biol. (2021) 9:789517. doi: 10.3389/fcell.2021.789517 34869391 PMC8635032

[95] Canonica GW Varricchi G Paoletti G Heffler E Virchow JC. Advancing precision medicine in asthma: evolution of treatment outcomes. J Allergy Clin Immunol. (2023) 152:835–40. doi: 10.1016/j.jaci.2023.07.009 37531979

[96] Bai D Sun T Lu F Shen Y Zhang Y Zhang B. Eupatilin suppresses ova-induced asthma by inhibiting nf-Kb and mapk and activating nrf2 signaling pathways in mice. Int J Mol Sci. (2022) 23(3):1582. doi: 10.3390/ijms23031582 35163503 PMC8836136

[97] Chen GH Song CC Pantopoulos K Wei XL Zheng H Luo Z. Mitochondrial oxidative stress mediated fe-induced ferroptosis via the nrf2-are pathway. Free Radic Biol Med. (2022) 180:95–107. doi: 10.1016/j.freeradbiomed.2022.01.012 35045311

[98] Liu C Wu X Bing X Qi W Zhu F Guo N. H1n1 influenza virus infection through nrf2-keap1-gclc pathway induces ferroptosis in nasal mucosal epithelial cells. Free Radic Biol Med. (2023) 204:226–42. doi: 10.1016/j.freeradbiomed.2023.05.004 37146698

[99] Liu J Xu Y Yan M Yu Y Guo Y. 18β-glycyrrhetinic acid suppresses allergic airway inflammation through nf-Kb and nrf2/ho-1 signaling pathways in asthma mice. Sci Rep. (2022) 12:3121. doi: 10.1038/s41598-022-06455-6 35210449 PMC8873505

[100] Zhao J O’Donnell VB Balzar S St Croix CM Trudeau JB Wenzel SE. 15-lipoxygenase 1 interacts with phosphatidylethanolamine-binding protein to regulate mapk signaling in human airway epithelial cells. Proc Natl Acad Sci USA. (2011) 108:14246–51. doi: 10.1073/pnas.1018075108 PMC3161579 21831839

[101] Nagasaki T Schuyler AJ Zhao J Samovich SN Yamada K Deng Y. 15lo1 dictates glutathione redox changes in asthmatic airway epithelium to worsen type 2 inflammation. J Clin Invest. (2022) 132(1):e151685. doi: 10.1172/jci151685 34762602 PMC8718153

[102] Wenzel SE Tyurina YY Zhao J St Croix CM Dar HH Mao G. Pebp1 wardens ferroptosis by enabling lipoxygenase generation of lipid death signals. Cell. (2017) 171:628–41.e26. doi: 10.1016/j.cell.2017.09.044 29053969 PMC5683852

[103] Li N Liu B Xiong R Li G Wang B Geng Q. Hdac3 Deficiency Protects against Acute Lung Injury by Maintaining Epithelial Barrier Integrity through Preserving Mitochondrial Quality Control. Redox Biol. (2023) 63:102746. doi: 10.1016/j.redox.2023.102746 37244125 PMC10199751

[104] Zhuang C Kang M Lee M. Delivery systems of therapeutic nucleic acids for the treatment of acute lung injury/acute respiratory distress syndrome. J Control Release. (2023) 360:1–14. doi: 10.1016/j.jconrel.2023.06.018 37330013

[105] Lv YW Du Y Ma SS Shi YC Xu HC Deng L. Proanthocyanidins attenuates ferroptosis against influenza-induced acute lung injury in mice by reducing ifn-Γ. Life Sci. (2023) 314:121279. doi: 10.1016/j.lfs.2022.121279 36526043

[106] Dong H Xia Y Jin S Xue C Wang Y Hu R. Nrf2 attenuates ferroptosis-mediated iir-ali by modulating tert and slc7a11. Cell Death Dis. (2021) 12:1027. doi: 10.1038/s41419-021-04307-1 34716298 PMC8556385

[107] Li Y Cao Y Xiao J Shang J Tan Q Ping F. Inhibitor of apoptosis-stimulating protein of P53 inhibits ferroptosis and alleviates intestinal ischemia/reperfusion-induced acute lung injury. Cell Death Differ. (2020) 27:2635–50. doi: 10.1038/s41418-020-0528-x PMC7429834 32203170

[108] He R Liu B Xiong R Geng B Meng H Lin W. Itaconate inhibits ferroptosis of macrophage via nrf2 pathways against sepsis-induced acute lung injury. Cell Death Discov. (2022) 8:43. doi: 10.1038/s41420-021-00807-3 35110526 PMC8810876

[109] Liu P Feng Y Li H Chen X Wang G Xu S. Ferrostatin-1 alleviates lipopolysaccharide-induced acute lung injury via inhibiting ferroptosis. Cell Mol Biol Lett. (2020) 25:10. doi: 10.1186/s11658-020-00205-0 32161620 PMC7045739

[110] Dong H Qiang Z Chai D Peng J Xia Y Hu R. Nrf2 inhibits ferroptosis and protects against acute lung injury due to intestinal ischemia reperfusion via regulating slc7a11 and ho-1. Aging (Albany NY). (2020) 12:12943–59. doi: 10.18632/aging.103378 PMC7377827 32601262

[111] Ma A Feng Z Li Y Wu Q Xiong H Dong M. Ferroptosis-related signature and immune infiltration characterization in acute lung injury/acute respiratory distress syndrome. Respir Res. (2023) 24:154. doi: 10.1186/s12931-023-02429-y 37301835 PMC10257327

[112] Zhang H Liu J Zhou Y Qu M Wang Y Guo K. Neutrophil extracellular traps mediate M(6)a modification and regulates sepsis-associated acute lung injury by activating ferroptosis in alveolar epithelial cells. Int J Biol Sci. (2022) 18:3337–57. doi: 10.7150/ijbs.69141 PMC9134924 35637949

[113] Chen C Zhang Z Tan F Meng F Lai L Chi X. Stabilizing mast cells improves acute lung injury after orthotopic liver transplantation via promotion of apoptosis in polymorphonuclear neutrophils. Am J Physiol Lung Cell Mol Physiol. (2021) 320:L266–l75. doi: 10.1152/ajplung.00046.2020 33174448

[114] Yang Y Ma Y Li Q Ling Y Zhou Y Chu K. Stat6 inhibits ferroptosis and alleviates acute lung injury via regulating P53/slc7a11 pathway. Cell Death Dis. (2022) 13:530. doi: 10.1038/s41419-022-04971-x 35668064 PMC9169029

[115] Liu X Zhang J Xie W. The role of ferroptosis in acute lung injury. Mol Cell Biochem. (2022) 477:1453–61. doi: 10.1007/s11010-021-04327-7 PMC8853161 35166985

[116] Dasgupta Q Jiang A Wen AM Mannix RJ Man Y Hall S. A human lung alveolus-on-a-chip model of acute radiation-induced lung injury. Nat Commun. (2023) 14:6506. doi: 10.1038/s41467-023-42171-z 37845224 PMC10579267

[117] Li X Chen J Yuan S Zhuang X Qiao T. Activation of the P62-keap1-nrf2 pathway protects against ferroptosis in radiation-induced lung injury. Oxid Med Cell Longev. (2022) 2022:8973509. doi: 10.1155/2022/8973509 35847598 PMC9277166

[118] Li X Duan L Yuan S Zhuang X Qiao T He J. Ferroptosis inhibitor alleviates radiation-induced lung fibrosis (Rilf) via down-regulation of tgf-B1. J Inflamm (Lond). (2019) 16:11. doi: 10.1186/s12950-019-0216-0 31160885 PMC6542066

[119] Ye LF Chaudhary KR Zandkarimi F Harken AD Kinslow CJ Upadhyayula PS. Radiation-induced lipid peroxidation triggers ferroptosis and synergizes with ferroptosis inducers. ACS Chem Biol. (2020) 15:469–84. doi: 10.1021/acschembio.9b00939 PMC7180072 31899616

[120] Li X Zhuang X Qiao T. Role of ferroptosis in the process of acute radiation-induced lung injury in mice. Biochem Biophys Res Commun. (2019) 519:240–5. doi: 10.1016/j.bbrc.2019.08.165 31493867

[121] Qiu H Cao S Xu R. Cancer incidence, mortality, and burden in China: A time-trend analysis and comparison with the United States and United Kingdom based on the global epidemiological data released in 2020. Cancer Commun (Lond). (2021) 41:1037–48. doi: 10.1002/cac2.12197 PMC8504144 34288593

[122] Siegel RL Miller KD Fuchs HE Jemal A. Cancer statistics, 2021. CA Cancer J Clin. (2021) 71:7–33. doi: 10.3322/caac.21654 33433946

[123] Lovly CM. Expanding horizons for treatment of early-stage lung cancer. N Engl J Med. (2022) 386:2050–1. doi: 10.1056/NEJMe2203330 35403840

[124] Tang Z Jiang W Mao M Zhao J Chen J Cheng N. Deubiquitinase usp35 modulates ferroptosis in lung cancer via targeting ferroportin. Clin Transl Med. (2021) 11:e390. doi: 10.1002/ctm2.390 33931967 PMC8087931

[125] Bebber CM Thomas ES Stroh J Chen Z Androulidaki A Schmitt A. Ferroptosis response segregates small cell lung cancer (Sclc) neuroendocrine subtypes. Nat Commun. (2021) 12:2048. doi: 10.1038/s41467-021-22336-4 33824345 PMC8024350

[126] Wang X Chen Y Wang X Tian H Wang Y Jin J. Stem cell factor sox2 confers ferroptosis resistance in lung cancer via upregulation of slc7a11. Cancer Res. (2021) 81:5217–29. doi: 10.1158/0008-5472.Can-21-0567 PMC8530936 34385181

[127] Zhang W Sun Y Bai L Zhi L Yang Y Zhao Q. Rbms1 regulates lung cancer ferroptosis through translational control of slc7a11. J Clin Invest. (2021) 131(22):e152067. doi: 10.1172/jci152067 34609966 PMC8592553

[128] Liang J Guan X Bao G Yao Y Zhong X. Molecular subtyping of small cell lung cancer. Semin Cancer Biol. (2022) 86:450–62. doi: 10.1016/j.semcancer.2022.05.010 35609720

[129] Ortega-Franco A Ackermann C Paz-Ares L Califano R. First-line immune checkpoint inhibitors for extensive stage small-cell lung cancer: clinical developments and future directions. ESMO Open. (2021) 6:100003. doi: 10.1016/j.esmoop.2020.100003 33450659 PMC7811117

[130] Sundarakrishnan A Chen Y Black LD Aldridge BB Kaplan DL. Engineered cell and tissue models of pulmonary fibrosis. Adv Drug Deliv Rev. (2018) 129:78–94. doi: 10.1016/j.addr.2017.12.013 29269274

[131] Podolanczuk AJ Thomson CC Remy-Jardin M Richeldi L Martinez FJ Kolb M. Idiopathic pulmonary fibrosis: state of the art for 2023. Eur Respir J. (2023) 61(4):2200957. doi: 10.1183/13993003.00957-2022 36702498

[132] Pei Z Qin Y Fu X Yang F Huo F Liang X. Inhibition of ferroptosis and iron accumulation alleviates pulmonary fibrosis in a bleomycin model. Redox Biol. (2022) 57:102509. doi: 10.1016/j.redox.2022.102509 36302319 PMC9614651

[133] Sun L He X Kong J Yu H Wang Y. Menstrual blood-derived stem cells exosomal mir-let-7 to ameliorate pulmonary fibrosis through inhibiting ferroptosis by sp3/hdac2/nrf2 signaling pathway. Int Immunopharmacol. (2024) 126:111316. doi: 10.1016/j.intimp.2023.111316 38056200

[134] Warren R Lyu H Klinkhammer K De Langhe SP. Hippo signaling impairs alveolar epithelial regeneration in pulmonary fibrosis. Elife. (2023) 12:e85092. doi: 10.7554/eLife.85092 37166104 PMC10208641

[135] Cao D Zheng J Li Z Yu Y Chen Z Wang Q. Acsl4 inhibition prevents macrophage ferroptosis and alleviates fibrosis in bleomycin-induced systemic sclerosis model. Arthritis Res Ther. (2023) 25:212. doi: 10.1186/s13075-023-03190-9 37884942 PMC10601156

[136] Cheng H Feng D Li X Gao L Tang S Liu W. Iron deposition-induced ferroptosis in alveolar type ii cells promotes the development of pulmonary fibrosis. Biochim Biophys Acta Mol Basis Dis. (2021) 1867:166204. doi: 10.1016/j.bbadis.2021.166204 34175430

[137] Wu X Jiang Y Li R Xia Y Li F Zhao M. Ficolin B secreted by alveolar macrophage exosomes exacerbates bleomycin-induced lung injury via ferroptosis through the cgas-sting signaling pathway. Cell Death Dis. (2023) 14:577. doi: 10.1038/s41419-023-06104-4 37648705 PMC10468535

[138] Tao N Li K Liu J. Molecular mechanisms of ferroptosis and its role in pulmonary disease. Oxid Med Cell Longev. (2020) 2020:9547127. doi: 10.1155/2020/9547127 32685102 PMC7338975

[139] Gong Y Wang N Liu N Dong H. Lipid peroxidation and gpx4 inhibition are common causes for myofibroblast differentiation and ferroptosis. DNA Cell Biol. (2019) 38:725–33. doi: 10.1089/dna.2018.4541 31140862

[140] Rashidipour N Karami-Mohajeri S Mandegary A Mohammadinejad R Wong A Mohit M. Where ferroptosis inhibitors and paraquat detoxification mechanisms intersect, exploring possible treatment strategies. Toxicology. (2020) 433-434:152407. doi: 10.1016/j.tox.2020.152407 32061663

[141] Yue D Zhang Q Zhang J Liu W Chen L Wang M. Diesel exhaust pm2.5 greatly deteriorates fibrosis process in pre-existing pulmonary fibrosis via ferroptosis. Environ Int. (2023) 171:107706. doi: 10.1016/j.envint.2022.107706 36565570

[142] Heyckendorf J Georghiou SB Frahm N Heinrich N Kontsevaya I Reimann M. Tuberculosis treatment monitoring and outcome measures: new interest and new strategies. Clin Microbiol Rev. (2022) 35:e0022721. doi: 10.1128/cmr.00227-21 35311552 PMC9491169

[143] Amaral EP Costa DL Namasivayam S Riteau N Kamenyeva O Mittereder L. A major role for ferroptosis in mycobacterium tuberculosis-induced cell death and tissue necrosis. J Exp Med. (2019) 216:556–70. doi: 10.1084/jem.20181776 PMC6400546 30787033

[144] Qiang L Zhang Y Lei Z Lu Z Tan S Ge P. A mycobacterial effector promotes ferroptosis-dependent pathogenicity and dissemination. Nat Commun. (2023) 14:1430. doi: 10.1038/s41467-023-37148-x 36932056 PMC10023711

[145] Gan B. Ferroptosis hijacking by mycobacterium tuberculosis. Nat Commun. (2023) 14:1431. doi: 10.1038/s41467-023-37149-w 36932073 PMC10023749

[146] Amaral EP Foreman TW Namasivayam S Hilligan KL Kauffman KD Barbosa Bomfim CC. Gpx4 regulates cellular necrosis and host resistance in mycobacterium tuberculosis infection. J Exp Med. (2022) 219(11):e20220504. doi: 10.1084/jem.20220504 36069923 PMC9458471

[147] Guan WJ Ni ZY Hu Y Liang WH Ou CQ He JX. Clinical characteristics of coronavirus disease 2019 in China. N Engl J Med. (2020) 382:1708–20. doi: 10.1056/NEJMoa2002032 PMC7092819 32109013

[148] Wu Z McGoogan JM. Characteristics of and important lessons from the coronavirus disease 2019 (Covid-19) outbreak in China: summary of a report of 72 314 cases from the chinese center for disease control and prevention. Jama. (2020) 323:1239–42. doi: 10.1001/jama.2020.2648 32091533

[149] Bednash JS Kagan VE Englert JA Farkas D Tyurina YY Tyurin VA. Syrian hamsters as a model of lung injury with sars-cov-2 infection: pathologic, physiologic, and detailed molecular profiling. Transl Res. (2022) 240:1–16. doi: 10.1016/j.trsl.2021.10.007 34740873 PMC8562047

[150] Jacobs W Lammens M Kerckhofs A Voets E Van San E Van Coillie S. Fatal lymphocytic cardiac damage in coronavirus disease 2019 (Covid-19): autopsy reveals a ferroptosis signature. ESC Heart Fail. (2020) 7:3772–81. doi: 10.1002/ehf2.12958 PMC7607145 32959998

[151] Wang Y Huang J Sun Y Stubbs D He J Li W. Sars-cov-2 suppresses mrna expression of selenoproteins associated with ferroptosis, endoplasmic reticulum stress and DNA synthesis. Food Chem Toxicol. (2021) 153:112286. doi: 10.1016/j.fct.2021.112286 34023458 PMC8139185

[152] Peleman C Van Coillie S Ligthart S Choi SM De Waele J Depuydt P. Ferroptosis and pyroptosis signatures in critical covid-19 patients. Cell Death Differ. (2023) 30:2066–77. doi: 10.1038/s41418-023-01204-2 PMC10482958 37582864

[153] Jankauskas SS Kansakar U Sardu C Varzideh F Avvisato R Wang X. Covid-19 causes ferroptosis and oxidative stress in human endothelial cells. Antioxidants (Basel). (2023) 12(2):326. doi: 10.3390/antiox12020326 36829885 PMC9952002

[154] Liu L Du J Yang S Zheng B Shen J Huang J. Sars-cov-2 orf3a sensitizes cells to ferroptosis via keap1-nrf2 axis. Redox Biol. (2023) 63:102752. doi: 10.1016/j.redox.2023.102752 37245288 PMC10202901

[155] Nuszkiewicz J Sutkowy P Wróblewski M Pawłowska M Wesołowski R Wróblewska J. Ferroptosis and sars-cov-2 infection. Antioxidants (Basel). (2023) 12(3):733. doi: 10.3390/antiox12030733 36978981 PMC10045478

[156] Simonis A Kreer C Albus A Rox K Yuan B Holzmann D. Discovery of highly neutralizing human antibodies targeting pseudomonas aeruginosa. Cell. (2023) 186:5098–113.e19. doi: 10.1016/j.cell.2023.10.002 37918395

[157] Deshpande R Zou C. Pseudomonas aeruginosa induced cell death in acute lung injury and acute respiratory distress syndrome. Int J Mol Sci. (2020) 21(15):5356. doi: 10.3390/ijms21155356 32731491 PMC7432812

[158] Dar HH Tyurina YY Mikulska-Ruminska K Shrivastava I Ting HC Tyurin VA. Pseudomonas aeruginosa utilizes host polyunsaturated phosphatidylethanolamines to trigger theft-ferroptosis in bronchial epithelium. J Clin Invest. (2018) 128:4639–53. doi: 10.1172/jci99490 PMC6159971 30198910

[159] Banthiya S Pekárová M Kuhn H Heydeck D. Secreted lipoxygenase from pseudomonas aeruginosa exhibits biomembrane oxygenase activity and induces hemolysis in human red blood cells. Arch Biochem Biophys. (2015) 584:116–24. doi: 10.1016/j.abb.2015.09.003 26361973

[160] Dar HH Anthonymuthu TS Ponomareva LA Souryavong AB Shurin GV Kapralov AO. A new thiol-independent mechanism of epithelial host defense against pseudomonas aeruginosa: inos/no(•) sabotage of theft-ferroptosis. Redox Biol. (2021) 45:102045. doi: 10.1016/j.redox.2021.102045 34167028 PMC8227829

[161] Vaillancourt M Galdino ACM Limsuwannarot SP Celedonio D Dimitrova E Broerman M. A compensatory rnase E variation increases iron piracy and virulence in multidrug-resistant pseudomonas aeruginosa during macrophage infection. PloS Pathog. (2023) 19:e1010942. doi: 10.1371/journal.ppat.1010942 37027441 PMC10115287

[162] Yang YQ Chen HP Wang Y Yin LM Xu YD Ran J. Considerations for use of acupuncture as supplemental therapy for patients with allergic asthma. Clin Rev Allergy Immunol. (2013) 44:254–61. doi: 10.1007/s12016-012-8321-3 22661215

[163] Dong M Wang WQ Chen J Li MH Xu F Cui J. Acupuncture regulates the balance of cd4(+) T cell subtypes in experimental asthma mice. Chin J Integr Med. (2019) 25:617–24. doi: 10.1007/s11655-018-3055-6 30519873

[164] Li B Yang L Peng X Fan Q Wei S Yang S. Emerging mechanisms and applications of ferroptosis in the treatment of resistant cancers. BioMed Pharmacother. (2020) 130:110710. doi: 10.1016/j.biopha.2020.110710 33568263

[165] Viswanathan VS Ryan MJ Dhruv HD Gill S Eichhoff OM Seashore-Ludlow B. Dependency of a therapy-resistant state of cancer cells on a lipid peroxidase pathway. Nature. (2017) 547:453–7. doi: 10.1038/nature23007 PMC5667900 28678785

[166] Liu W Zhou Y Duan W Song J Wei S Xia S. Glutathione peroxidase 4-dependent glutathione high-consumption drives acquired platinum chemoresistance in lung cancer-derived brain metastasis. Clin Transl Med. (2021) 11:e517. doi: 10.1002/ctm2.517 34586745 PMC8473645

[167] Zhang K Wu J Zhao X Qin J Xue Y Zheng W. PRussian blue/calcium peroxide nanocomposites-mediated tumor cell iron mineralization for treatment of experimental lung adenocarcinoma. ACS Nano. (2021) 15:19838–52. doi: 10.1021/acsnano.1c07308 34851083

[168] Li Y Yang J Gu G Guo X He C Sun J. Pulmonary delivery of theranostic nanoclusters for lung cancer ferroptosis with enhanced chemodynamic/radiation synergistic therapy. Nano Lett. (2022) 22:963–72. doi: 10.1021/acs.nanolett.1c03786 35073699

[169] Niu X Chen L Li Y Hu Z He F. Ferroptosis, necroptosis, and pyroptosis in the tumor microenvironment: perspectives for immunotherapy of sclc. Semin Cancer Biol. (2022) 86:273–85. doi: 10.1016/j.semcancer.2022.03.009 35288298

[170] Wang W Green M Choi JE Gijón M Kennedy PD Johnson JK. Cd8(+) T cells regulate tumour ferroptosis during cancer immunotherapy. Nature. (2019) 569:270–4. doi: 10.1038/s41586-019-1170-y PMC6533917 31043744

[171] Liu T Liu W Zhang M Yu W Gao F Li C. Ferrous-supply-regeneration nanoengineering for cancer-cell-specific ferroptosis in combination with imaging-guided photodynamic therapy. ACS Nano. (2018) 12:12181–92. doi: 10.1021/acsnano.8b05860 30458111

[172] Hsieh CH Hsieh HC Shih FS Wang PW Yang LX Shieh DB. An innovative nrf2 nano-modulator induces lung cancer ferroptosis and elicits an immunostimulatory tumor microenvironment. Theranostics. (2021) 11:7072–91. doi: 10.7150/thno.57803 PMC8171079 34093872

[173] Liu T Yang Q Zheng H Jia H He Y Zhang X. Multifaceted roles of a bioengineered nanoreactor in repressing radiation-induced lung injury. Biomaterials. (2021) 277:121103. doi: 10.1016/j.biomaterials.2021.121103 34478930

[174] Dai Y Shan W Yang Q Guo J Zhai R Tang X. Biomarkers of iron metabolism facilitate clinical diagnosis in mycobacterium tuberculosis infection. Thorax. (2019) 74:1161–7. doi: 10.1136/thoraxjnl-2018-212557 PMC6902069 31611342

[175] Wang P Pradhan K Zhong XB Ma X. Isoniazid metabolism and hepatotoxicity. Acta Pharm Sin B. (2016) 6:384–92. doi: 10.1016/j.apsb.2016.07.014 PMC5045547 27709007

[176] Maio N Lafont BAP Sil D Li Y Bollinger JM Jr. Krebs C. Fe-S cofactors in the sars-cov-2 rna-dependent rna polymerase are potential antiviral targets. Science. (2021) 373:236–41. doi: 10.1126/science.abi5224 PMC8892629 34083449

[177] Han Y Zhu J Yang L Nilsson-Payant BE Hurtado R Lacko LA. Sars-cov-2 infection induces ferroptosis of sinoatrial node pacemaker cells. Circ Res. (2022) 130:963–77. doi: 10.1161/circresaha.121.320518 PMC8963443 35255712

